# Age-dependent removal of Atg9-containing vesicle accumulations in motoneuron disease models by physical exercise

**DOI:** 10.1186/s40035-025-00524-2

**Published:** 2025-12-16

**Authors:** Alexander Veh, Melissa Ewald, Vinicius da Cruz Neris Geßner, Neha Jadhav Giridhar, Amy-Jayne Hutchings, Christian Stigloher, Beyenech Binotti, Katrin Gertrud Heinze, Patrick Lüningschrör

**Affiliations:** 1 Institute of Clinical Neurobiology, University Hospital Würzburg, Versbacher Str. 5, 97078 Würzburg, Germany; 2 Rudolf Virchow Center for Integrative and Translational Bioimaging, Julius-Maximilians-Universität Würzburg (JMU), Josef-Schneider-Str. 2, 97080 Würzburg, Germany; 3 Imaging Core Facility, Biocenter, Julius-Maximilians-Universität Würzburg (JMU), 97074 Würzburg, Germany; 4 Heidelberg University Biochemistry Center (BZH), 69120 Heidelberg, Germany

**Keywords:** Atg9, Plekhg5, Autophagy, Motoneuron disease, Axon, Physical exercise

## Abstract

**Background:**

Atg9-containing vesicles are enriched in synapses and undergo cycles of exo- and endocytosis similarly to synaptic vesicles, thereby linking presynaptic autophagy to neuronal activity. Dysfunction of presynaptic autophagy is a pathophysiological mechanism in motoneuron disease (MND), which leads to impaired synaptic integrity and function. Here, we asked whether boosting neuronal activity by physical exercise modulates the cellular and motor phenotypes of *Plekhg5*-deficient mice, an MND model with defective presynaptic autophagy.

**Methods:**

To characterize the vesicle accumulations in *Plekhg5*-deficient mice, we performed immunohistochemical staining, electron microscopy, and super-resolution imaging. Following voluntary running wheel exercise, we quantified the histopathological changes within the spinal cord and at neuromuscular junctions using an unbiased machine-learning approach. Additionally, we analyzed the motor performance of the animals by measuring their grip strength. To assess changes in the autophagic flux upon physical exercise in vivo, we utilized mRFP-GFP-LC3 expressing mice. The presence of Atg9-containing vesicle clusters in SOD1^G93A^ was analyzed to examine the relevance of this pathological feature in a second MND model.

**Results:**

We found marked accumulations of Atg9-containing vesicles at presynaptic sites of *Plekhg5*-deficient mice, which could be cleared by four weeks of voluntary running wheel exercise in young but surprisingly not in aged Plekhg5-deficient mice. However, physical exercise in aged mice led to synaptic vesicle sorting into the Atg9-containing vesicle accumulations without their removal. In line with these findings, short-term voluntary exercise triggered motoneuron autophagy in young but not old mice. Pointing to a broader role of Atg9-containing vesicles in the pathophysiology of MND, we also found Atg9-containing vesicle accumulations in SOD1^G93A^ mice, a well-established ALS model. Strikingly, physical exercise in presymptomatic SOD1^G93A^ mice resulted in a reduction of the vesicle accumulations.

**Conclusions:**

Our data highlight the essential role of Atg9 in presynaptic autophagy and suggest that boosting autophagy by physical exercise provides a tool to maintain presynaptic function at the early but not late stages of *Plekhg5*-associated MND and possibly amyotrophic lateral sclerosis.

**Supplementary Information:**

The online version contains supplementary material available at 10.1186/s40035-025-00524-2.

## Background

Autophagy in neurons contributes to the maintenance of global proteostasis [1, 2], but also mediates the turnover of pre- and postsynaptic components [3–6]. Interaction of the autophagy machinery with distinct synaptic proteins and organelles enables the removal of aged and defective components from synapses [3]. Vice versa, synaptic activity triggers neuronal autophagy [6]. During synaptic activity, neurotransmitters are released from synaptic vesicles (SVs) by exocytosis. Subsequently, the SV membranes are recaptured by endocytosis for recycling [7]. During this SV cycle, aged and damaged SVs need to be recognized for removal to ensure the long-term maintenance of presynaptic function. While the recycling of SVs occurs at a basal level, enhanced neuronal activity increases protein turnover to match the higher demand and recycling of vesicles [8]. Consecutive usage of muscles is linked to increased neuronal activity of motoneurons (MN). Physical exercise, such as running, increases MN firing patterns [9].

Presynaptic Atg9-containing vesicles undergo activity-dependent endo- and exocytosis cycles similar to SVs [10]. Disruption of endocytosis causes the accumulation of Atg9 and a defective activity-dependent autophagy [10]. Presynaptic Atg9-containing vesicles co-purify with SVs, but only a subset of SVs carries Atg9, indicating that Atg9-containing vesicles represent a distinct heterogeneous vesicle population [11]. Functionally, Atg9 is a lipid scramblase that mediates autophagosomal membrane expansion [12]. Depletion of Atg9 in vitro results in the absence of isolation membrane formation [13]. Nervous system-specific deletion of *Atg9* in mice causes axon-specific lesions leading to neurodegeneration and a motor phenotype [14]. While Atg9 is required for autophagic vesicle formation, the small GTPase Rab26 mediates the targeted autophagy of SVs. Rab26 is enriched on a subset of SVs and binds to Atg16L, specifically in its GTP-bound form, enabling it to direct SVs to phagophores [15]. The guanine exchange factor Plekhg5 (Pleckstrin homology domain containing family G) regulates the activity of Rab26 [16]. Mutations in the human *PLEKHG5* gene have been linked to different forms of lower motoneuron disease (MND) [17]. Initially, a single homozygous missense mutation was identified in an individual extended consanguineous African family diagnosed with a unique form of autosomal recessive lower MND with childhood-onset, distal spinal muscular atrophy 4 [18]. More recently, additional *PLEKHG5* mutations were frequently identified in patients suffering from autosomal recessive intermediate Charcot-Marie-Tooth disease, distal spinal muscular atrophy, and distal hereditary motor neuropathy [17, 19]. Depletion of *Plekhg5* in mice leads to an MND with marked vesicle accumulations at MN terminals, in peripheral nerves, and within the spinal cord [16, 20].

While disruption of SV recycling itself causes neurodegeneration, it remains unclear how elevated neuronal activity impacts disease progression in different MNDs. Here, we utilized voluntary physical exercise to induce presynaptic autophagy in motoneurons. Induction of neuronal activity by physical exercise opens an in vivo approach to increase vesicle turnover, and allows the investigation of Atg9-containing vesicle clusters in wild-type and *Plekhg5*-deficient mice. Using this approach, our study aims to explore the influence of neuronal activity on presynaptic autophagy and its role in the pathophysiology of MND.

## Materials and methods

### Mouse handling and voluntary physical exercise

All mice used in this study are listed in Table S1. Mice were handled according to the institutional guidelines of the University Clinic Wuerzburg and the German federal law of animal protection. Mice were kept at constant room temperature and humidity at a 12/12-h light condition with food and water offered *ad libitum*. Mice aged 3 and 12 months were split into sedentary and running groups for voluntary physical exercise. Exercising mice were individually placed within a new cage containing a running wheel (1800/50, Ugo Basile, Germonio, Italy) and left undisturbed to perform voluntary physical exercise for either four hours or four weeks consecutively. Mice from the sedentary group were individualized as well and housed without running wheels. Physical exercise was evaluated based on a daily recording of running wheel revolutions. Mice which performed less than 800 revolutions within four hours were excluded from the experiments. Mice which performed less than 800 revolutions daily on three consecutive days were excluded from the experiments. All mice had access to water and food ad libitum during the entire time. Grip-strength was measured for all mice before and after four weeks of physical exercise. Measurements were carried out on two consecutive days. Grip strength (93153, Ametek, Berlin, Germany) was measured at least 3 times per mouse.

### Immunohistochemistry for muscles

Mice were sacrificed, tibialis anterior (TA) and gastrocnemius (GAS) muscles removed, fibers separated and fixed for two hours in 4% paraformaldehyde (PFA). Fibers were washed three times with PBS, and once with 0.1 mol/L glycine for 15 min. Samples were incubated in 10% donkey serum with 0.3% Triton X-100 in TBS-T for two hours at room temperature (RT). Three subsequent washing steps with TBS-T containing 0.1% Triton X-100 were performed and samples were incubated with primary antibodies in blocking solution for one day at 4 °C. Samples were washed three times, incubated with secondary antibodies at RT for 2 h, and washed with PBS. Fibers were carefully separated and evenly distributed on an object slide before mounting with FlourSave (Merck, 345789, Darmstadt, Germany). To visualize postsynaptic acetylcholine receptors (AChRs), α-bungarotoxin (BTX) conjugated to Alexa-488 from Invitrogen (B13422, Dreieich, Germany) was used. Secondary antibodies were obtained from Jackson Immuno-Research Laboratories. Primary antibodies are listed in Table S2. Secondary antibodies are listed in Table S3.

### Immunohistochemistry for spinal cord sections

Mice were euthanized for 5 min with CO_2_ and trans-cardially perfused with 4% PFA. Spinal cords were removed after perfusion, post-fixed for two hours with 4% PFA, and sections cut at a Leica VT1000S Vibratome with 40-µm thickness. Free-floating sections were washed three times with PBS, once with 0.1 mol/L glycine for 15 min and once with ammonium-acetate for 30 min. Subsequently, samples were blocked in 10% donkey serum with 0.3% Triton X-100 in TBS-T for two hours at RT and incubated with primary antibodies for two days at 4 °C. Samples were washed three times, incubated with secondary antibodies at RT for two hours, and washed thoroughly with PBS. Afterwards, samples were mounted with FlourSave (Merck, 345789). Secondary antibodies were obtained from Jackson Immuno-Research Laboratories. DAPI (Sigma-Aldrich, D9542-5MG, Darmstadt, Germany) was used for nucleic acid staining. Primary antibodies are listed in Table S2. Secondary antibodies are listed in Table S3.

### Quantification of neuromuscular junction (NMJ) integrity

Images of NMJs were randomized and individually analyzed for shape, integrity and balloon-like accumulations at the presynapses. Only presynaptic staining colocalized with postsynaptic marker BTX was analyzed. NMJ shape was categorized as unaffected, fragmented with partial loss of presynaptic staining compared to corresponding postsynaptic staining, and denervated with more than 90% of presynaptic marker gone. Balloon-like structures were counted as presynaptic accumulation with an area of more than 3 µm diameter.

### Quantification of clusters in spinal cord sections

All spinal cord images were randomized and analyzed in an unbiased way. The Deepflash 0.2.3 learning algorithm was used to identify fluorescence signals [21]. Spinal cord sections were observed under a confocal scanning microscope (Olympus FV1000) and raw images were captured and then converted to 8 bits and scaled to 1024 × 1024 pixels resolution by ImageJ. 20 × magnification images were stitched with the ImageJ stitching plugin. Spinal cord samples with low abundance of targeted signal were imaged with 40 × magnification. For evaluation of the Deepflash data, five image training data sets were created manually for model generation by experts. Ground truth estimation was performed with masks from five experts. Different models were created for respective experiments and fluorescence patterns. Model performance metrics were based on the inbuild dice score for semantic segmentation, with retraining of models until a dice score of at least 0.7 was reached. Furthermore, resulting masks from model training were cross-checked by experts to ensure model quality. Resulting images were frequently controlled by random sampling and sporadic false positive values resulting from cell body detection were manually removed. Spinal cord gray matter and NMJ areas were measured using free-hand sections in ImageJ. Spinal cord sections were counted from one side including the ventral horn up to the central canal. Generated image masks were analyzed with the particle analyze function, ignoring particles with a size below 10 pixel and outside the gray matter area. Measured particles were normalized to the respective gray matter area and resolution and displayed as number per area. Graphic creation and statistics were performed with OriginPro 2021b Academic. Outliers were removed upon statistical significance by Grubbs’ outlier test.

### Quantification of autophagosomes and autolysosomes

Images were processed as described above. MNs and NMJs were manually traced for area calculation. Corresponding masks from GFP and RFP were colocalized, and double-positive signals were counted as autophagosomes. Autophagosome numbers were subtracted from the total RFP^+^ signals and the remaining RFP^+^ signals were counted as autolysosomes. Signals were normalized to respective areas. Outliers were removed upon statistical significance by Grubbs’ outlier test.

### Membrane fractionation

Whole spinal cord tissues were dissected and immediately frozen in liquid nitrogen. Fractionation was performed following the protocol from Wirths [22]. Briefly, tissue was homogenized in homogenization buffer and centrifuged for 10 min at 1000 *g*. The buffer contained 0.32 mol/L sucrose, 5 mmol/L HEPES, protease inhibitor (5892970001, Millipore Sigma, Darmstadt, Germany) and phosphatase inhibitor (4906837001, Millipore Sigma). The supernatant was collected (Input) and centrifuged at 17,000 *g* for 30 min. The pellet was washed with PBS and centrifuged at 17,000 g for 30 min. The supernatant was transferred into polycarbonate tubes (252240, Beranek, Nußloch, Germany) and centrifuged at 100,000 *g* for 1 h. The supernatant was collected (Cytosol), and the pellet was washed with PBS. Finally, the washed pellet from the 100,000 *g* centrifugation (100 k) was resuspended in PBS. The input from 3–4 spinal cord homogenates was pooled to obtain sufficient material for the 100 k pellet. Protein yield was determined by the Bradford assay. All centrifugation steps were carried out at 4 °C.

### Western blots

Equal amounts of protein were separated by SDS-PAGE, and transferred to PVDF membranes (1620177, Bio-Rad, Dreieich, Germany) (120 V, 45 min, 4 °C). Membranes were blocked in TBS-T with 5% milk powder for 2 h at RT, probed with primary antibodies overnight at 4 °C, and incubated with horseradish peroxidase-conjugated secondary antibodies for 1 h at RT. Membranes were washed three times in TBS-T for 15 min and incubated for 5 min with developer reagents (Immobilon, WBKLS0500, Darmstadt, Germany). Primary antibodies used for Western blot analysis are listed in Table S4. Peroxidase-conjugated secondary antibodies against mouse (715–005-150) and rabbit (711–005-152) were obtained from Jackson Immuno-Research Laboratories (Cambridgeshire, UK). Peroxidase-conjugated secondary antibody against goat (AP180P) was obtained from Merck.

### Electron microscopy

For ultrastructure analysis, mice were transcardially perfused following a modified Forssman perfusion protocol [23]. Two fixation solutions were subsequently used, the first containing 1.5% PFA with 1.5% glutaraldehyde and the second containing 3% PFA, 3% glutaraldehyde and 0.05% picric acid in water. Mice were slowly perfused with 75 mL fixative one and 100 mL fixative two. Afterward, tissue was dissected and post-fixed in 4% PFA with 4% glutaraldehyde for two hours at 4 °C. Samples were washed with 0.1 mol/L phosphate buffer and contrasted with 2% osmium tetroxide for 2.5 h at 4 °C. The staining and embedding protocol was modified after Mulisch and Welsch [24]. Briefly, fixed samples were washed in 0.1 mol/L phosphate buffer, incubated in 0.5% aqueous uranyl acetate overnight, washed and dehydrated in serial ethanol concentrations from 50% to 100%. Dehydrated samples were placed two times in propylene oxide for 30 min each at RT, followed by a 1:1 mix of propylene oxide and epoxy resin overnight. The samples were then embedded in epoxy resin. Cured samples were sliced to 70-nm sections and imaged under a transmission electron microscope (JEOL JEM-2100, Freising, Germany) at 200 kV with a TVIPS F416 digital camera.

### High-resolution direct stochastic optical reconstruction microscopy (dSTORM) imaging

Mice were sacrificed, spinal cord tissue removed, and immediately frozen in tissue tack (Sakura Finetek, 4583, Umkirch, Germany) at − 80 °C. Samples were cut with a cryostat (Leica CM 1950) at 5 µm thickness and mounted on coverslips coated with poly-*L*-lysine. The sections were fixed with 4% PFA for 15 min at RT, followed by incubation with 0.1 mol/L glycine for 15 min. Samples were blocked in 10% donkey serum with 0.3% Triton X-100 in TBS-T for two hours at RT and incubated with primary antibodies for one day at 4 °C. After rinsing with PBS, secondary antibodies were applied for two hours, followed by three washing steps with PBS. The dual-color dSTORM images were acquired using a Zeiss Observer Z.1 inverted light microscope (Carl Zeiss AG) at the Rudolf Virchow Center for Integrative and Translational Bioimaging (Würzburg, Germany). The design was adapted for single-molecule localization microscopy (SMLM) as shown in Table S5. The blinking buffer contained 125 mmol/L cysteamine hydrochloride (Sigma, M6500), 20 mmol/L D-glucose (Sigma, G7528), 0.55 mg/mL glucose oxidase (Roth, 60281), and 0.011 mg/mL catalase (Sigma, C1345) in PBS, with pH adjusted to 7.7 using a 5 mol/L KOH solution [25]. The order of acquisition was important to avoid unnecessary bleaching. First, A647 was acquired, followed by Cy3. The frames of the dSTORM images were reconstructed using the ThunderSTORM Plugin for ImageJ [26, 27], generating super-resolved images with a pixel size of 102 nm. Detailed camera setups and image processing specifications are listed in Table S6.

### Cell culture of primary mouse MNs and optogenetic stimulation

Spinal MNs from embryonic mice were isolated and cultured as previously described [28, 29]. Briefly, spinal cords from E13 embryos were dissected and incubated for 15 min in 0.05% trypsin in Hank’s balanced salt solution. Cells were triturated and incubated in Neurobasal medium, supplemented with 100 µg/mL penicillin–streptomycin-glutamine (Gibco, 10378016) on Nunclon plates (Thermo Fisher Scientific, 150350, Dreieich, Germany) pre-coated with antibodies against the p75 NGF receptor (MLR2, a kind gift from Robert Rush, Flinders University, Adelaide, Australia) for 45 min. After washing with Neurobasal medium, the remaining MNs were recovered with a depolarization solution (0.8% NaCl, 35 mmol/L KCl and 2 mmol/L CaCl_2_). Cells were subsequently collected in the MN medium (2% horse serum, 1 × B-27 in Neurobasal medium with Glutamax). Before plating, MNs were transduced with lentiviral vectors for expression of ChR2-YFP and RFP-LC3. Cells were plated on four-well dishes (Greiner Bio-One, 627170, Schwerte, Germany) pre-coated with poly-ornithine/laminin (Sigma-Aldrich, L2020-1MG). MNs were cultured with the neurotrophic factor BDNF (10 ng/mL) following a medium change every second day.

Stimulation experiments were performed on day 7. Control samples were protected from light at least one hour before fixation. Stimulation samples were placed beneath a blue light source (470 nm, 10% laser intensity) and exposed for 2 min with 0.2 Hz followed by a recovery time of 8 min in an incubator. Exposure was repeated three times and MNs were subsequently fixed in 4% PFA for 15 min.

### Immunocytochemistry of cultured MNs

Fixed MNs were washed once with TBS-T and blocked with 10% horse serum in TBS-T for one hour. The samples were incubated in primary antibody solution containing TBS-T overnight at 4 °C. Afterwards, samples were washed three times with PBS and incubated with secondary antibody solution in PBS for one hour. MNs were washed three times with PBS and mounted in FlourSave. Alexa-488-, Cy3- and Cy5-conjugated secondary antibodies were obtained from Jackson Immuno-Research Laboratories. Primary antibodies are listed in Table S2.

## Results

### Atg9-containing vesicles accumulate in axon terminals of *Plekhg5*-deficient mice

**Fig. 1:**
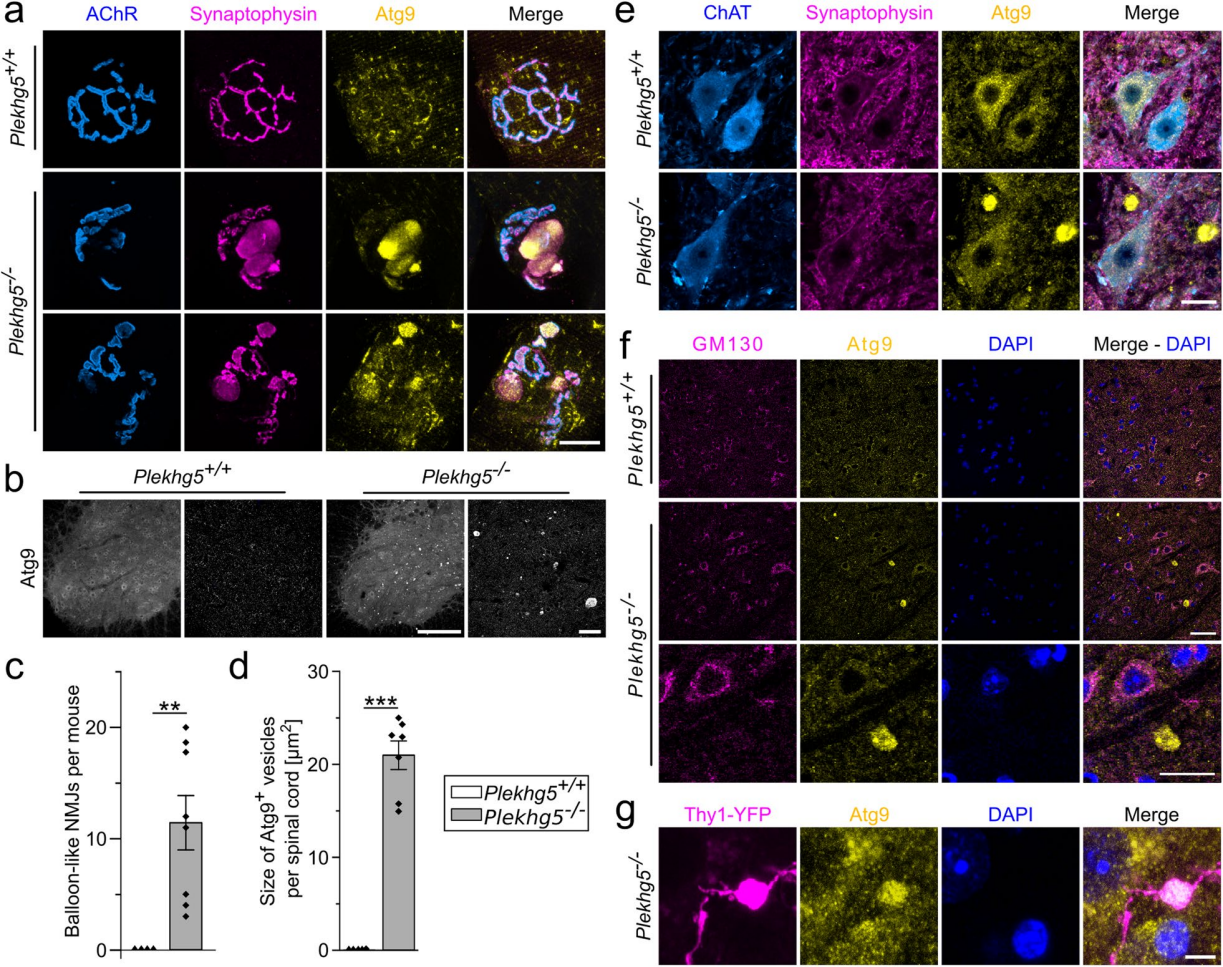
Atg9-containing vesicles accumulate in MN axon terminals and axons of *Plekhg5*-deficient mice. **a** Atg9-containing vesicles accumulate in axon terminals of motoneurons (MN) at neuromuscular junctions (NMJs) within the tibialis anterior muscle of *Plekhg5*^*−/−*^ mice. Synaptophysin and Atg9 were labeled by immunohistochemical staining. The postsynaptic membrane was visualized by staining of the AChRs with fluorophore-conjugated Bungarotoxin (BTX). Scale bar, 20 µm. **b** Atg9-containing vesicles accumulate in the spinal cords of *Plekhg5*^*−/−*^ mice. Immunofluorescence of Atg9 in the lumbar spinal cord sections from *Plekhg5*^+*/*+^ and *Plekhg5*^*−/−*^mice. Scale bar, ventral horn 100 µm, magnification 50 µm. **c** Quantification of balloon-like structures in *Plekhg5*^+*/*+^ and *Plekhg5*^*−/−*^ mice at NMJs in skeletal muscle. *Plekhg5*^+*/*+^ *n* = 4; *Plekhg5*^*−/−*^* n* = 8. One sample *T*-test. **d** Quantification of Atg9^+^ cluster sizes between *Plekhg5*^+*/*+^ and *Plekhg5*^*−/−*^mice. Ventral horn spinal cord sections were analyzed. *Plekhg5*^+*/*+^ *n* = 5; *Plekhg5*^*−/−*^* n* = 7. One sample *T*-test. **e** Atg9-containing vesicle clusters are absent from MN somata. Scale bar, 20 µm. **f** Atg9-containing vesicle clusters are negative for the Golgi marker GM130. Lumbar spinal cord cross-sections from 3-month-old wild-type and *Plekhg5*-deficient mice. Scale bar, upper panels 40 µm, lower panels 10 µm. **g** Atg9-containing vesicle clusters localize in axons. Spinal cord cross-sections of *Plekhg5*^*−/−*^* Thy1::YFP* mice stained for Atg9, GFP and DAPI. Scale bar, 5 µm. Data are shown as mean ± SEM; **P < 0.01; ***P < 0.001. Images from at least 3 biological replicates

Depletion of Plekhg5 results in marked accumulation of SVs in MN terminals due to impaired clearance by Rab26-mediated autophagy [16]. Besides SVs, Atg9-containing vesicles (Atg9^+^ vesicles) are reported to cycle at the presynaptic terminal coupled to neuronal activity [10]. To address whether the pathophysiological mechanism also involves Atg9, we analyzed the localization of Atg9 in MNs of wild-type and *Plekhg5*-deficient mice. We visualized Atg9 and the presynaptic SV marker Synaptophysin by immunohistochemical staining and the corresponding postsynaptic membranes by fluorophore-conjugated BTX, which binds to AChR. In wild-type animals, we detected individual Atg9^+^ vesicles in presynaptic terminals of MNs with some overlap to Synaptophysin (Fig. 1a), which is consistent with previous studies [10, 11]. In contrast, Atg9^+^ vesicles markedly accumulated in swollen, “balloon-like” presynaptic terminals in *Plekhg5*-deficient mice (Fig. 1a, c). In addition, we detected numerous Atg9^+^ vesicle clusters in spinal cord cross-sections of *Plekhg5*-deficient mice (Fig. 1b, d). These Atg9^+^ vesicle clusters were outside of MN somata, as shown by co-staining with choline acetyltransferase (ChAT) (Fig. 1e). Moreover, the Golgi marker GM130 was absent from the vesicle accumulations (Fig. 1f), confirming that Atg9 does not accumulate at the Golgi apparatus as shown for the depletion of AP4 [30]. We hypothesized that the Atg9^+^ clusters represent axon terminals of neurons, which project into the spinal cord. To visualize individual axons, we crossed *Plekhg5*-deficient mice with Thy1-YFP mice, leading to a sparse expression of YFP in individual neurons. Using this strategy, we detected Atg9^+^ vesicle accumulations in axons within the spinal cord, confirming the neuronal origin and axonal localization of the Atg9^+^ vesicle clusters (Fig. 1g). In summary, our data show the accumulation of Atg9^+^ vesicles upon *Plekhg5* deficiency within the spinal cord and presynaptic MN terminals in skeletal muscles.

### Physical exercise reduces the clustering of Atg9-containing vesicles in young, but not aged *Plekhg5*-deficient mice

Based on the activity-coupled cycling of Atg9^+^ vesicles [10], we investigated whether exercise-induced neuronal activity could change the accumulation of Atg9^+^ vesicles in the spinal cord. Therefore, we analyzed the Atg9^+^ accumulations in the gray matter of spinal cord sections upon four hours of voluntary physical exercise using a deep learning segmentation pipeline [21]. After four hours of voluntary exercise, we observed only minor, non-significant differences in the size and number of Atg9^+^ clusters (Fig. S1), suggesting that four hours are not sufficient to modulate the accumulations.

**Fig. 2:**
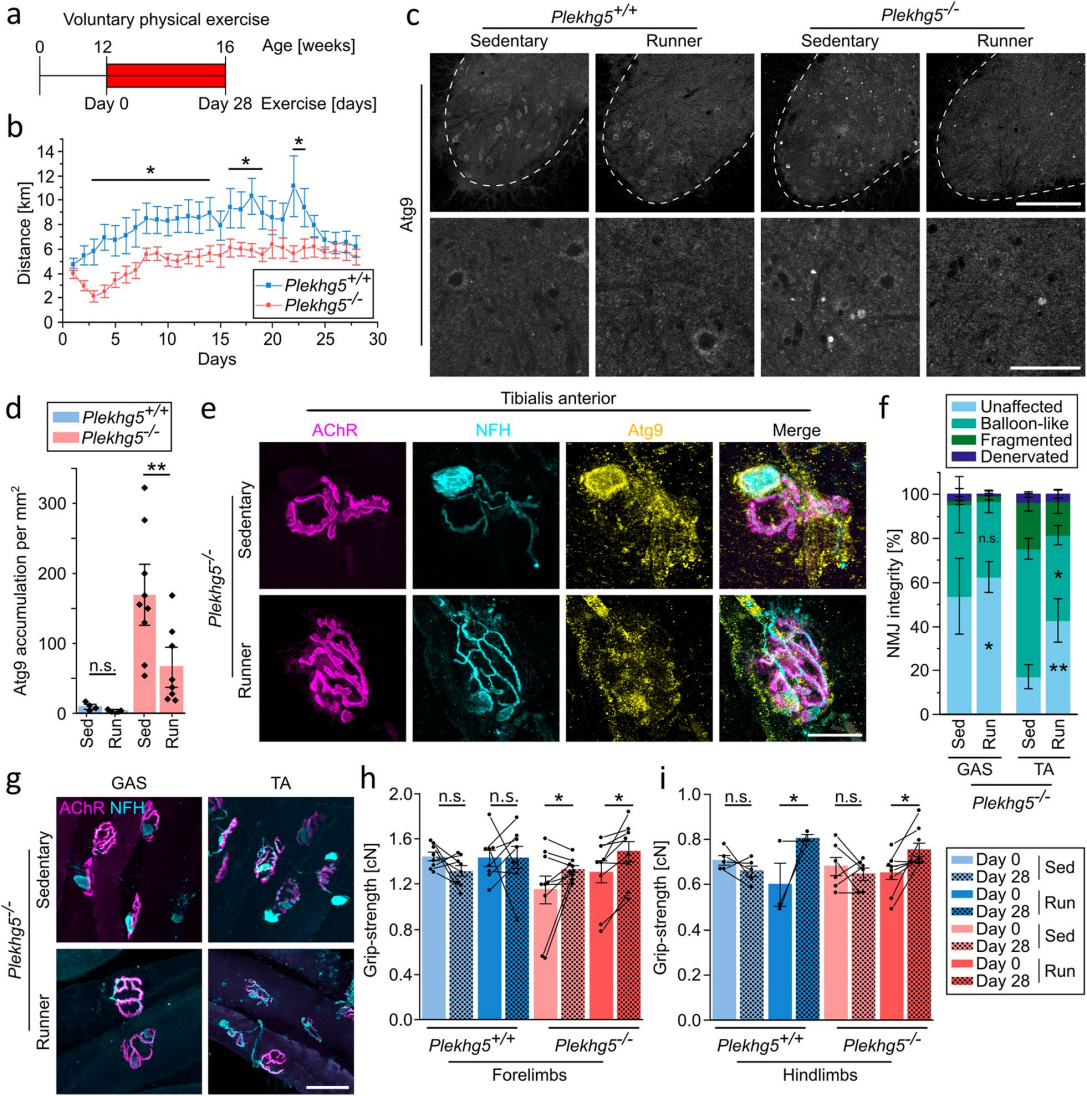
Physical exercise improves the motor and cellular phenotypes of 3-month-old *Plekhg5*-deficient mice. **a** Scheme of the paradigm for voluntary physical exercise in 3-month-old mice. **b** The 3-month-old *Plekhg5*-deficient mice ran less distance daily than control animals during the 4 weeks of voluntary physical exercise. *Plekhg5*^+*/*+^ *n* = 9; *Plekhg5*^*−/−*^* n* = 8. Two-way ANOVA; Holm-Bonferroni multiple comparison test. **c** Physical exercise reduced the number of Atg9-contaning vesicle clusters in the spinal cord of *Plekhg5*^*−/−*^mice. Scale bars, 200 µm for upper panels, 60 µm for lower panels. **d** Quantification of Atg9-containing vesicle cluster in sedentary (Sed) and running (Run) 3-month-old *Plekhg5*^+*/*+^ and *Plekhg5*^*−/−*^ mice. Each data point represents the mean number of clusters from at least 7 cross-sections from each mouse. *Plekhg5*^+*/*+^ Sed, *n* = 4; *Plekhg5*^+*/*+^ Run, *n* = 4; *Plekhg5*^*−/−*^ Sed, *n* = 9; *Plekhg5*^*−/−*^ Run, *n* = 8. Two-way ANOVA; Holm-Bonferroni multiple comparison test. **e** Atg9 accumulates in balloon-like structures at NMJs from tibialis anterior muscle in both sedentary and running mice. NFH and Atg9 immunofluorescence and BTX-coupled fluorophore in 3-month-old *Plekhg5*-deficient mice. Scale bar, 20 µm. **f** Quantification of NMJ integrity from sedentary and running 3-month-old *Plekhg5*-deficient mice showing increased number of unaffected NMJs after exercise. GAS-Sed, *n* = 5; GAS-Run, *n* = 7; TA-Sed, *n* = 5; TA-Run, *n* = 7. Each data point represents the mean from at least 15 NMJs. Two-way ANOVA; Holm-Bonferroni multiple comparison test. **g** Increased number of unaffected NMJs after 4 weeks of physical exercise in tibialis anterior muscles. NMJs in the gastrocnemius (GAS) and tibialis anterior (TA) muscles from 3-month-old *Plekhg5*^*−/−*^ mice. Presynaptic membrane visualized by immunofluorescence, postsynaptic membrane by BTX-coupled fluorophore. Scale bar, 50 µm. **h, i** Physical exercise improved grip strength of 3-month-old *Plekhg5*-deficient mice. Each data point represents the mean of 3–5 single grip-strength measurements per mouse. Connected dots represent identical mice prior to and after exercise. **h** Forelimbs; Sed *Plekhg5*^+*/*+^ mice, *n* = 8; Run *Plekhg5*^+*/*+^ mice, *n* = 8; Sed *Plekhg5*^*−/−*^ mice, *n* = 9; Run *Plekhg5*^*−/−*^ mice, *n* = 8. **i** Hindlimbs; Sed *Plekhg5*^+*/*+^ mice, *n* = 5; Run *Plekhg5*^+*/*+^ mice, *n* = 3; Sed *Plekhg5*^*−/−*^ mice, *n* = 6; Run *Plekhg5*^*−/−*^ mice, *n* = 8. Two-way repeated measures ANOVA; Holm-Bonferroni multiple comparison test. Data are shown as mean ± SEM; n.s., not significant; *P < 0.05, **P < 0.01; all representative images are taken from at least 3 biological replicates

Thus, we investigated the impact of continuous physical exercise in *Plekhg5*-deficient mice. We analyzed the clustering of Atg9^+^ vesicles in 3-month-old mice, which had access to a running wheel for four consecutive weeks in comparison to sedentary animals (Fig. 2a). Compared to their wild-type counterparts, *Plekhg5*-deficient mice performed less daily exercise, yet both groups performed consistently throughout the entire four weeks (Fig. 2b). After four weeks of exercise, we detected a significantly reduced number of Atg9^+^ accumulations in spinal cord cross-sections of *Plekhg5*-deficient mice (Fig. 2c, d). Notably, physical exercise improved the integrity of NMJs in the TA and GAS muscles of *Plekhg5*-deficient mice, resulting in an increase of unaffected NMJs without vesicle accumulation and a decrease of “balloon-like” NMJs (Fig. 2e–g). This beneficial effect was more pronounced in the TA muscle (Fig. 2f). The TA muscle, comprised of more type IIb and IIX fast-twitching muscle fibers, exerts higher activity, which likely results in an increased turnover of Atg9^+^ clusters [31, 32]. However, we did not observe any differences in the size and quantity of Atg9^+^ clusters per NMJs after physical exercise (Fig. S2a, c, e). To study the impact of physical exercise on motor performance, we measured the grip strength before and after four weeks of physical exercise. Grip strength was improved in the forelimbs and hindlimbs of *Plekhg5*-deficient mice after four weeks of physical exercise (Fig. 2h, i).

**Fig. 3:**
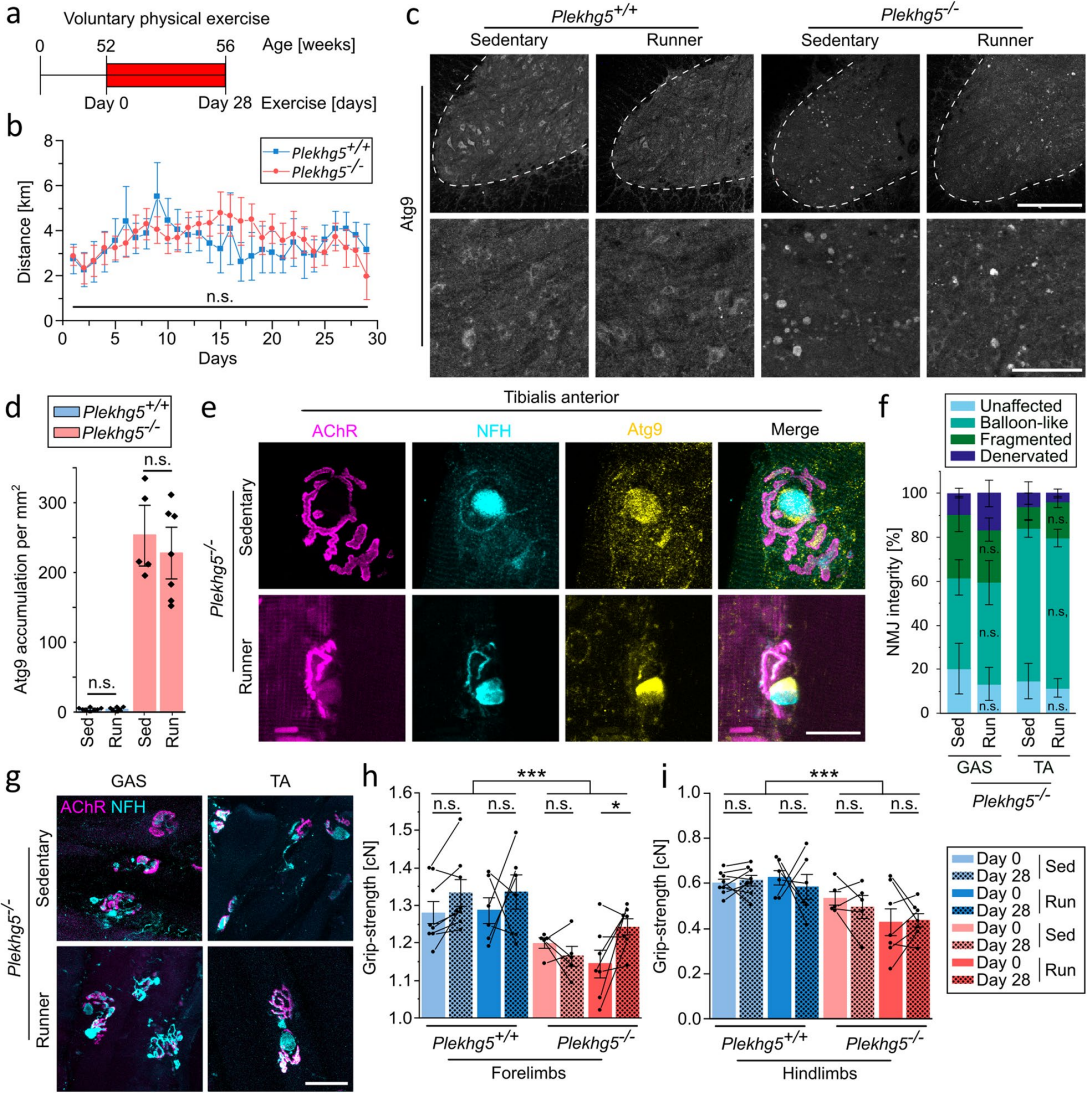
Physical exercise has no impact on the cellular phenotype of 12-month-old *Plekhg5*-deficient mice. **a** Scheme of the paradigm for voluntary exercise in 12-month-old mice. **b** The 12-month-old *Plekhg5*-deficient and control mice ran comparable distances daily during 4 weeks of voluntary physical exercise. *Plekhg5*^+*/*+^ *n* = 6; *Plekhg5*^*−/−*^* n* = 7. Two-way ANOVA; Holm-Bonferroni multiple comparison test. **c** Physical exercise had no impact on the number of Atg9-containg vesicle clusters in spinal cord cross-sections of 12-month-old *Plekhg5*-deficint mice. Scale bar, 200 µm for upper panels, 60 µm for lower panels. **d** Quantification of Atg9^+^ vesicle clusters with exercise in 12-month-old *Plekhg5*^+*/*+^ and *Plekhg5*^*−/−*^ mice. Each data point represents the mean number of clusters from at least 7 cross sections. Sed *Plekhg5*^+*/*+^ mice, *n* = 8; Run *Plekhg5*^+*/*+^ mice, *n* = 6; Sed *Plekhg5*^*−/−*^ mice, *n* = 5; Run *Plekhg5*^*−/−*^ mice, *n* = 7. Two-way ANOVA; Holm-Bonferroni multiple comparison test. **e** Atg9 accumulates in balloon-like structures in NMJs from tibialis anterior muscles in both sedentary and running *Plekhg5*-deficient mice. Scale bar, 20 µm. **f** Quantification of NMJ integrity from sedentary and running 12-month-old *Plekhg5*-deficient mice shows no improvement with exercise. GAS-Sed, *n* = 5; Gas-Run, *n* = 7; TA-Sed, *n* = 5; TA-Run, *n* = 7. Each data point represents the mean from at least 15 NMJs each. Two-way ANOVA; Holm-Bonferroni multiple comparison test. **g** Presynaptic balloon-like swellings remain similar in NMJs from 12-month-old *Plekhg5*^*−/−*^ mice. Presynaptic membrane visualized by NFH immunofluorescence, postsynaptic membrane by BTX-coupled fluorophore. Scale bar, 50 µm. **h, i** Physical exercise improved the grip-strength in forelimbs (**h**) but not hindlimbs (**i**) of 12-month-old *Plekhg5*-deficient mice. Disease progression is visible by overall reduced grip-strength in *Plekhg5*-deficient mice. Each data point represents the mean of 3–5 single grip-strength measurements per animal. Sed *Plekhg5*^+*/*+^ mice, *n* = 8; Run *Plekhg5*^+*/*+^ mice, *n* = 6; Sed *Plekhg5*^*−/−*^ mice, *n* = 5; Run *Plekhg5*^*−/−*^ mice, *n* = 7. Two-way repeated measures ANOVA; Holm-Bonferroni multiple comparison test. Data are shown as mean ± SEM; n.s, not significant; *P < 0.05, ***P < 0.001; all representative images are taken from at least 3 biological replicates

We next wondered whether exercise in older mice with a more advanced disease progression might similarly counteract the phenotype. *Plekhg5*-deficient mice develop hindlimb clasping at around 12 months with progressive hindlimb paralysis at around 15 months of age [16]. Therefore, we subjected 12-month-old mice to four weeks of voluntary physical exercise (Fig. 3a). Both wild-type and *Plekhg5*-deficient mice performed comparable amounts of exercise (Fig. 3b). To our surprise, four weeks of voluntary exercise did not affect the number of Atg9^+^ clusters in 12-month-old spinal cords from *Plekhg5*-deficient mice (Fig. 3c, d). In aged mice, the NMJs from both TA and GAS muscles showed progressive degeneration without improvement after four weeks (Fig. 3e–g). Furthermore, we could not detect any changes in Atg9^+^ clusters within NMJs between sedentary and exercising mice in the TA (Fig. 3e) or GAS muscles (Fig. S2b). The size and quantity of Atg9^+^ clusters per NMJ remained unchanged after physical exercise (Fig. S2d, f). Correlating with the reduced number of unaffected NMJs, we found an overall reduced grip strength in *Plekhg5*-deficient mice (Fig. 3h, i). Nevertheless, four weeks of physical exercise still improved the motor performance in the forelimbs, leading to an elevated grip strength (Fig. 3h). However, *Plekhg5* deficiency primarily affects the hindlimbs with sparse accumulation of Atg9^+^ clusters in the cervical spinal cord sections [16]. In summary, our data show that physical exercise provides a paradigm to counteract presynaptic dysfunction in *Plekhg5*-deficient mice at an early age.

### Atg9^+^ cluster sorting remains functional despite clearing deficits at a later age

**Fig. 4:**
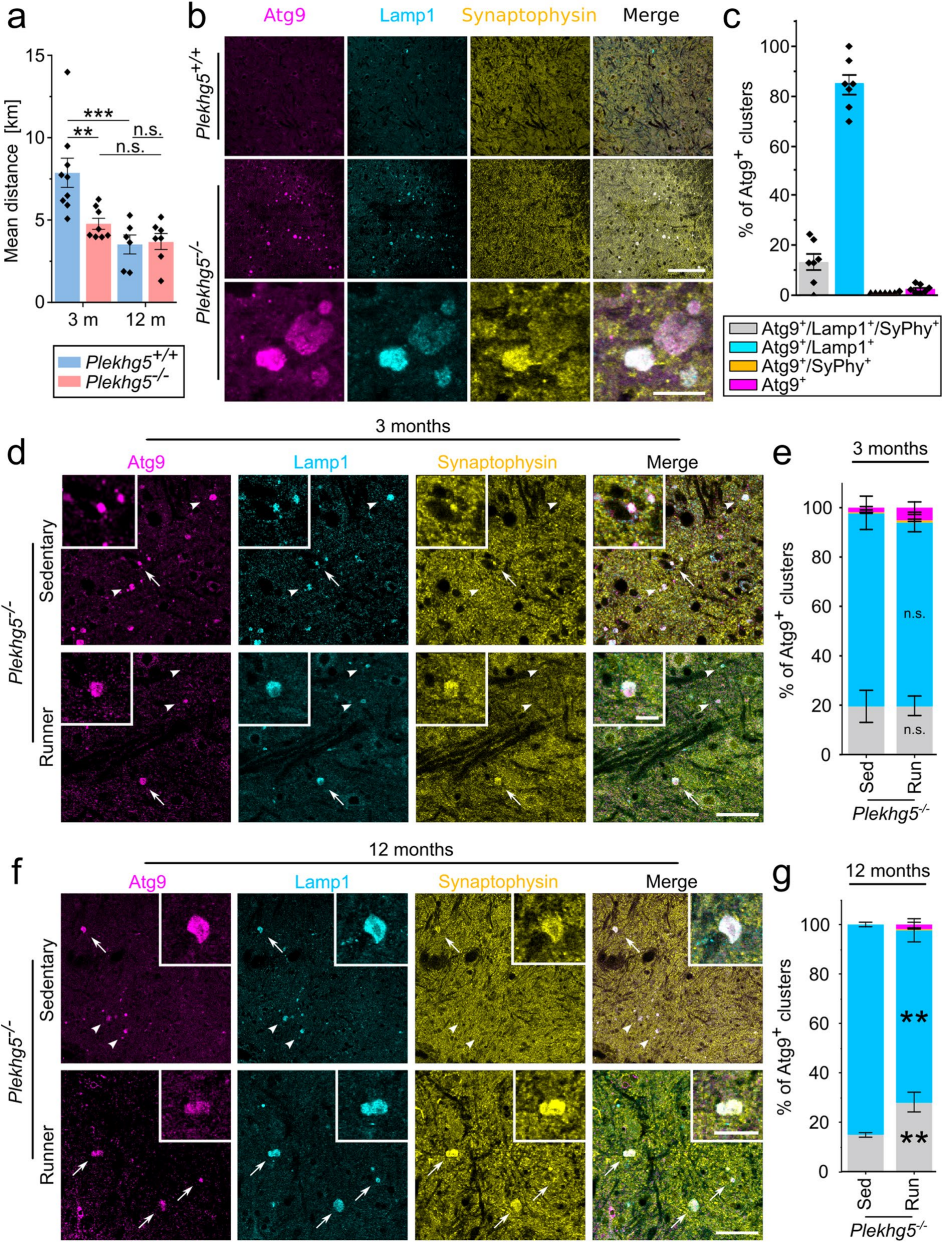
Sorting of synaptic vesicles into Atg9^+^ clusters continuous in 12-month-old *Plekhg5*-deficient mice **a** Comparison of the mean distance of running per mouse within 4 weeks. 3-month *Plekhg5*^+*/*+^ mice, *n* = 9; 3-month *Plekhg5*^*−/−*^ mice, *n* = 8; 12-month *Plekhg5*^+*/*+^ mice, *n* = 6; 12-month *Plekhg5*^*−/−*^ mice, *n* = 7. Two-way ANOVA; Holm-Bonferroni multiple comparison test. **b** Immunohistochemical labeling showing that Atg9^+^ clusters are primarily Lamp1^+^ with a minor population being positive for both Lamp1 and Synaptophysin. Spinal cord cross-sections from wild-type and *Plekhg5*-deficient mice. Scale bar, overview 50 µm, inset 10 µm. **c** Quantification of Atg9^+^ clusters positive for either Lamp1 or Synaptophysin from *Plekhg5*-deficient mice. Each data point represents the mean from seven spinal cord sections, *n* = 7. Data are given as percentage to the total amount of Atg9^+^ clusters. **d** Atg9-containing vesicle clusters sparsely colocalize with synaptic vesicles in 3-month-old *Plekhg5*-deficient mice. Atg9, Lamp1, and Synaptophysin immunoreactivity in spinal cord cross-sections. Arrows show triple positive staining; arrowheads show Atg9^+^ Lamp1^+^ clusters. Scale bar, 40 µm (main image) and 10 µm (magnified views). **e** The proportion of Atg9^+^ clusters co-localizing with either Lamp1 or Synaptophysin did not change upon physical exercise in 3-month-old mice. Means per mouse were calculated from 5 spinal cord sections. Sed *n* = 9; Run *n* = 8. Two-way ANOVA; Holm-Bonferroni multiple comparison test. **f** Atg9-containing vesicle clusters colocalized with synaptic vesicles increase upon physical exercise in 12-month-old mice. Immunofluorescence of Atg9, Lamp1 and Synaptophysin in spinal cord cross-sections. Arrows show triple positive staining; arrowheads show Atg9^+^ Lamp1^+^ clusters. Scale bar, 40 µm (main image) and 20 µm (magnified views). **g** Increased number of Atg9^+^Lamp1^+^Synaptophysin^+^ triple-positive vesicle clusters in the lumbar spinal cord cross-sections of *Plekhg5*^*−/−*^ mice after 4 weeks of voluntary physical exercise. Means per mouse were calculated from 5 spinal cord sections. Sed *n* = 5; Run *n* = 7. Two-way ANOVA; Holm-Bonferroni multiple comparison test. Data are shown as mean ± SEM; n.s., not significant; **P < 0.01; ***P < 0.001; all representative images are taken from at least 3 biological replicates

The inability to clear Atg9^+^ clusters with increased age led to the question of whether the removal of Atg9^+^ clusters was directly coupled to the performed exercise. To address this question, we analyzed the distances each mouse traveled per day (Fig. 4a). Whereas the three-month-old *Plekhg5*-deficient mice traveled significantly shorter distances than their wild-type counterpart, the average distances traveled by *Plekhg5*-deficient mice did not further decline at 12 months of age. Thus, *Plekhg5*-deficient mice ran comparable distances at both ages. These data rule out that aged mice failed to clear the Atg9^+^ clusters due to shorter distances traveled.

Despite the inability to clear Atg9^+^ clusters with progressive age, we wondered whether Atg9^+^ clusters reached a terminal, dysfunctional stage over time. Therefore, we analyzed the composition of Atg9^+^ clusters using immunohistochemistry. Based on previous work [11], we stained for the endo/lysosomal marker Lamp1 and the SV marker Synaptophysin. The majority of Atg9^+^ clusters stained positive for the late endosomal/lysosomal marker Lamp1, while only a minor subset was positive for the SV marker Synaptophysin (Fig. 4b, c). This finding aligns with the previously reported heterogeneity between SV and Atg9^+^ vesicle populations [11]. At the ultrastructural level, the vesicle clusters appeared as accumulations of individual, densely packed vesicles (Fig. S3a). Next, we further characterized the Atg9^+^ clusters, which overlapped with the late endosomal/lysosomal marker Lamp1. To address whether the Atg9^+^ Lamp1^+^ clusters represent a homogenous population with individual vesicles carrying both Atg9 and Lamp1 or a heterogenous population with individual vesicles carrying either Atg9 or Lamp1, we performed super-resolution microscopy (dSTORM). Using dSTORM, we detected individual ring-like structures, which carried both Atg9 and Lamp1 with a similar size range as the vesicles we detected by electron microscopy (Fig. S3b).

To determine whether age influences the Atg9^+^ cluster composition, we compared the Atg9^+^ clusters of 3- and 12-month-old mice with versus without physical exercise. While we did not observe any difference within 3-month-old mice (Fig. 4d, e), the proportion of the Atg9^+^ clusters positive for Synaptophysin significantly increased after exercise in 12-month-old mice (Fig. 4f, g). These findings suggest that physical exercise increases the sorting of SVs into existing Atg9^+^ clusters. While the overall reduction of Atg9^+^ clusters with exercise prevented the detection of vesicle composition changes in 3-month-old mice, sorting of SVs into the Atg9^+^ clusters is still ongoing with age, although its removal is no longer possible.

### Physical exercise induces MN autophagy in young but not aged mice

Neuronal activity requires the cycling of SVs and the subsequent removal of damaged vesicles through the autophagy machinery [6]. Based on the enhanced cycling of SVs during elevated neuronal activity [7], we investigated if and how physical exercise induces MN autophagy in young and aged mice. Due to the previously described age-dependent decline of autophagy biogenesis [33], we hypothesized that the failure to remove the Atg9-containing vesicles might result from impaired autophagy in aged mice.

To analyze changes in the autophagy levels, we crossed *Plekhg5*-deficient mice with mRFP-GFP-LC3 (Light chain 3) expressing mice. Fusion between autophagosomes and lysosomes leads to the acidification of the autolysosomes, which quenches the GFP signal [34]. Therefore, the tandem reporter enables differentiation between autophagosomes (RFP^+^GFP^+^) and autolysosomes (RFP^+^GFP^−^). This strategy allows quantification of the autophagic flux, which is particularly important due to the rapid turnover of autophagosomes in neurons [35]. First, we validated the expression of LC3-RFP-GFP in NMJs and MN somata. In MN somata, we mostly detected RFP^+^GFP^−^ vesicles, with only very few RFP^+^GFP^+^ vesicles (Fig. S4a). In contrast, comparable numbers of RFP^+^GFP^+^ and RFP^+^GFP^−^ vesicles were detectable within axon terminals at NMJs (Fig. S4b). These data align with the previously reported spatial separation of the autophagic process in neurons. Autophagosome biogenesis mostly occurs in distal axons, followed by maturation during retrograde transport and fusion with lysosomes at the somata [16, 36, 37]. We confirmed the presence of autophagosomes and the absence of lysosomes at MN axon terminals by electron microscopy. Vice versa, autophagosomes were hardly detectable in MN somata, while lysosomes were readily detectable (Fig. S4c).

**Fig. 5:**
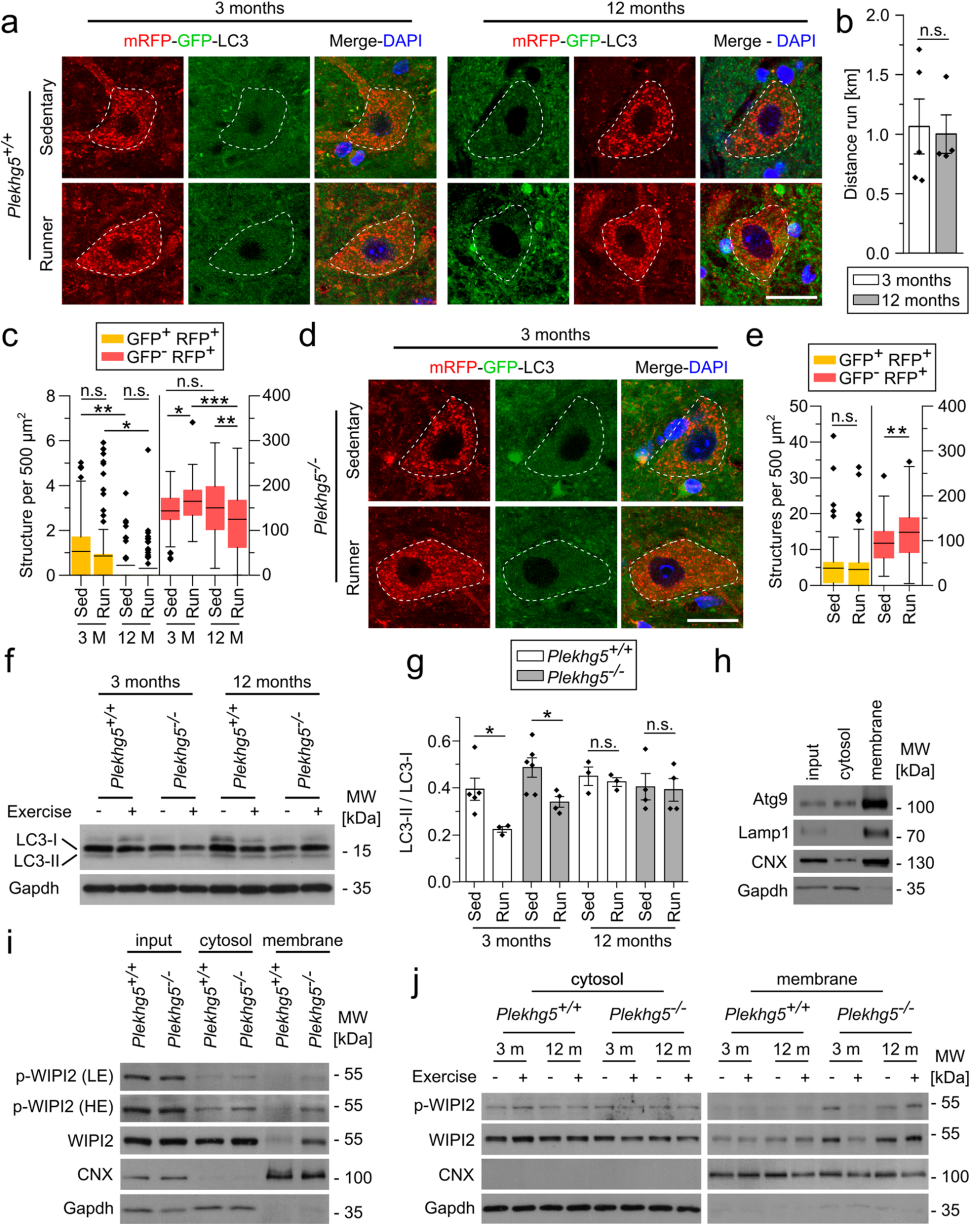
Short-term physical exercise increases autophagy in wild-type and *Plekhg5*-deficient mice. **a** Increased number of RFP^+^ puncta in somata of exercising 3-month-old mice and reduced RFP^+^ number within somata from 12-month-old wild-type mice. Immunofluorescence of GFP and RFP linked to LC3 with the nucleus labeled by DAPI. Scale bar, 20 µm. **b** Comparable distances of running during 4 h of voluntary physical exercise between 3- and 12-month-old wildtype mice. 3 months *n* = 5; 12 months *n* = 4. Two-sample *T*-test. **c** Quantification of GFP^+^ and RFP^+^ puncta numbers in MN somata from 3- and 12-month-old wildtype mice. Boxplot with mean, whiskers represent data points within 1.5 times the interquartile range from lower and upper quartiles. 3 months Sed, *n* = 70; 3 months Run, *n* = 81; 12 months Sed, *n* = 64; 12 months Run, *n* = 81. Two-way ANOVA; Holm-Bonferroni multiple comparison test. **d** Increased numbers of RFP^+^ signal in somata of exercising 3-month-old *Plekhg5*-deficient mice. Scale bar, 20 µm. **e** Quantification of GFP^+^ and RFP^+^ numbers in MN somata from 3-month-old *Plekhg5*-deficient mice. Boxplot with mean, whiskers represent data points within 1.5 times the interquartile range from lower and upper quartiles. Sed, *n* = 84; Run, *n* = 65. Two-way ANOVA; Holm-Bonferroni multiple comparison test. **f, g** Western blots and quantification depicting reduced levels of LC3-II in 3-month-old mice upon physical exercise. Whole spinal cord samples were analyzed. 3-month-old mice: Sed *Plekhg5*^+*/*+^ *n* = 5; Run *Plekhg5*^+*/*+^ *n* = 3; Sed *Plekhg5*^*−/−*^* n* = 6; Run *Plekhg5*^*−/−*^* n* = 4. 12-month-old mice: Sed *Plekhg5*^+*/*+^ *n* = 3; Run *Plekhg5*^+*/*+^ *n* = 3; Sed *Plekhg5*^*−/−*^* n* = 4; Run *Plekhg5*^*−/−*^* n* = 4. Two-way ANOVA; Holm-Bonferroni multiple comparison test. **h** Western blots showing enrichment of membrane proteins in the membrane pellet after differential centrifugation. Whole spinal cord lysates were fractionated, and the corresponding input, cytosolic, and membrane fractions were analyzed. **i** Western blots showing increased levels of phosphorylated WIPI2 and total WIPI2 in the membrane fraction of 3-month-old *Plekhg5*-deficient mice. Inputs were loaded alongside the cytosolic and membrane fractions. p-WIPI2 is shown with low exposure (LE) and high exposure (HE). Calnexin (CNX) and Gapdh were used as loading controls. **j** Western blots of cytosolic and membrane fractions from exercising and sedentary wild-type and *Plekhg5*-deficient mice. CNX and Gapdh were used as loading controls. Data are shown as mean ± SEM; n.s., not significant; *P < 0.05, *P < 0.01, ***P < 0.001; all representative images are taken from at least 3 biological replicates

Based on these findings, we used the number of autophagosomes and autolysosomes in MN somata as a readout to assess changes in the autophagy levels upon physical exercise. We subjected 3-month and 12-month-old mRFP-GFP-LC3 wild-type mice to four hours of voluntary physical exercise and analyzed the number of RFP^+^GFP^+^ and RFP^+^GFP^−^ vesicles using an unbiased machine learning approach (Fig. 5a). Mice from both cohorts performed comparable amounts of physical exercise (Fig. 5b). Whereas the number of autolysosomes in 3-month-old mice increased upon physical exercise, we observed a decrease in 12-month-old mice, demonstrating impairment of autophagy induction in aged mice (Fig. 5c). Furthermore, we observed significantly fewer autophagosomes in 12-month-old mice, suggesting reduced basal autophagy levels in aged mice. The number of autophagosomes did not change upon physical exercise in 3- and 12-month-old mice, most likely due to the rapid autophagic flux.

To confirm that autophagy induction depends on neuronal activity in a cell-autonomous manner, we expressed ChR2-YFP and RFP-LC3 in cultured primary mouse MNs. We analyzed the number of autophagosomes within axons after light stimulation and found an increased number of both SVs and autophagosomes (Fig. S4d–g). Furthermore, the number of autophagosomes carrying SVs as cargo also increased with blue light stimulation (Fig. S4g).

Next, we asked whether physical exercise also induced autophagy in 3-month-old *Plekhg5*-deficient mice. Therefore, we analyzed the number of autophagosomes and autolysosomes in MN somata from sedentary and exercising mice (Fig. 5d, e). In line with previous work [16], we detected a lower number of autolysosomes in sedentary *Plekhg5*-deficient mice compared to sedentary wildtype mice. Similar to wild-type mice, we found no change in the number of autophagosomes but an increased number of autolysosomes after physical exercise, strongly indicating that neuronal activity provides a mechanism to bypass the autophagy defects in *Plekhg5*-deficient mice. To corroborate our results biochemically, we examined the endogenous level of LC3 by Western blot analysis of spinal cord lysates (Fig. 5f, g). In the 3-month-old wild-type and *Plekhg5*-deficient mice, we detected a modest but robust decrease in LC3-II upon physical exercise. Notably, this decrease in LC3-II upon physical exercise was not detectable in 12-month-old mice.

The age-dependent autophagy decline in neurons has been linked to WIPI2B (WD repeat domain, phosphoinositide interacting 2B) [33]. Ectopically expressed WIPI2B restores autophagosome biogenesis in aged neurons [33]. This rescue requires the dynamic phosphorylation of WIPI2. Therefore, we performed membrane fractionation of spinal cords from 3-month-old wild-type and *Plekhg5*-deficient mice and analyzed the levels of WIPI2 and its phosphorylated form by Western blot (Fig. 5h–j). After homogenization and differential centrifugation of the tissue, we detected a clear enrichment of membrane proteins in the 100,000 *g* centrifugation pellet (membrane pellet), confirming the successful separation of the cytosolic and membrane fractions (Fig. 5h). Within the membrane fraction obtained from *Plekhg5*-deficient mice, we found an increased amount of phosphorylated-WIPI2 (p-WIPI2) compared to the wildtype (Fig. 5i). Interestingly, the total WIPI2 showed a comparable increase, suggesting an accumulation of WIPI2B in its phosphorylated form at isolation membranes. To assess whether the WIPI2 levels changed upon physical exercise, we analyzed the membrane fractions of sedentary and running mice at 3 and 12 months (Fig. 5j). Whereas no striking differences in the membrane levels of WIPI2 were detectable in the wildtype control samples, we observed a marked reduction of p-WIPI2 and WIPI2 levels in 3-month-old *Plekhg5*-deficient mice upon physical exercise (Fig. 5j). In 12-month-old animals, no decrease in the WIPI2 accumulation was apparent upon physical exercise. In the cytosolic fractions, we did not observe any striking differences. To further corroborate our findings, we also analyzed the levels of Atg9 in the membrane fraction. In line with our immunohistochemical data, we found a decrease of Atg9 in 3-month-old but not 12-month-old *Plekhg5*-deficient mice upon physical exercise (Fig. S4h).

In summary, these data indicate that short-term physical exercise accelerates the autophagic flux in MNs of 3-month-old mice, reflected by increased autolysosomes at the MN somata and reduced LC3-II in spinal cord lysates. Interestingly, physical exercise also triggered autophagy in *Plekhg5*-deficient mice, strongly indicating that Plekhg5 mediates SV turnover under basal conditions, uncoupled from neuronal activity, leading to the removal of Atg9^+^ clusters. Furthermore, our data point to WIPI2B as a major target to bypass the Plekhg5 deficiency-related autophagy impairment by physical exercise. While 3-month-old *Plekhg5*-deficient mice showed reduced WIPI2 levels following exercise, the effect was not apparent in 12-month-old mice, suggesting that WIPI2 accumulation upon Plekhg5 depletion can be reversed by neuronal activity.

### Atg9-containing vesicle accumulations are present in SOD1^G93A^ mice

**Fig. 6:**
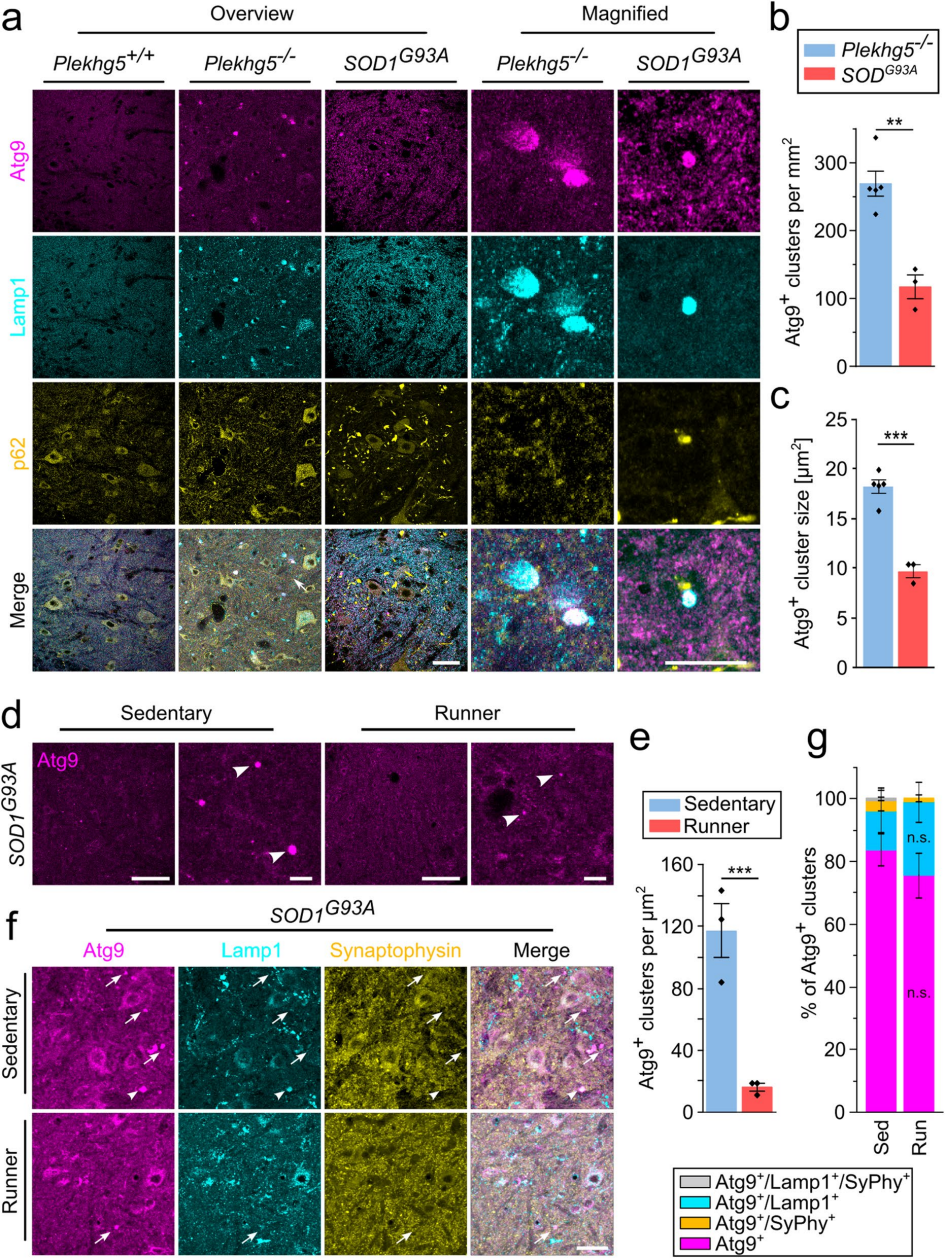
Reduction of Atg9^+^ clusters by physical exercise represents a broader mechanism found in ALS-linked *SOD1*-overexpressing mice. **a** Immunohistochemical staining showing that the Atg9^+^ vesicle clusters are present in SOD1 mice but negative for p62. Scale bars: 40 µm (overview) and 20 µm (magnified views). **b** *SOD1*-transgenic mice carry less Atg9^+^ clusters compared to *Plekhg5*-deficient mice. Each data point represents the mean from 7 spinal cord sections. *Plekhg5*^*−/−*^* n* = 5, *SOD1 n* = 3. Two sample *T*-test. **c** Quantification of Atg9 vesicle cluster size in spinal cord sections from *Plekhg5*-deficient and *SOD1* transgenic mice. Each data point represents the mean from 7 spinal cord sections. *Plekhg5*^*−/−*^* n* = 5, *SOD1*^*G93A*^* n* = 3. Two sample *T*-test. **d** Atg9-containing vesicle clusters are reduced upon physical exercise in 3-month-old SOD1^G93A^ mice. Arrowheads show Atg9 clusters. Scale bars: 50 µm (overview), 25 µm (magnified views). **e** Quantification of Atg9 clusters in sedentary and exercising *SOD1*^*G93A*^ mice. Each dot represents the mean from at least 7 spinal cord sections. Sedentary *n* = 3, Runner *n* = 3. Two sample *T*-test. **f** Immunohistochemical staining with Atg9, Lamp1 and Synaptophysin antibodies. Arrows indicate Atg9^+^ clusters negative for Lamp1 and Synaptophysin. Arrowheads indicate Atg9^+^Lamp1^+^ clusters. Scale bar, 30 µm. **g** Quantification of Atg9^+^ clusters colocalizing with either Lamp1, Synaptophysin or both. At least 5 spinal cords were analyzed per mouse. Sed *n* = 3; Run *n* = 3. Two sample *T*-test. Data are shown as mean ± SEM; n.s., not significant; **P < 0.01, ***P < 0.001; all representative images are taken from at least 3 biological replicates.

Next, we asked whether the clustering of Atg9^+^ vesicles represents a general hallmark of presynaptic dysfunction in MND. Therefore, we analyzed the distribution of Atg9^+^ clusters in spinal cord sections of SOD1^G93A^ mice, a well-established ALS model. Notably, we found Atg9^+^ vesicle clusters in spinal cord cross-sections from 8-month-old SOD1^G93A^ mice (Fig. 6a), albeit with a lower frequency and a smaller size compared to the *Plekhg5*-deficient mice (Fig. 6b, c). In contrast to the *Plekhg5*-deficient mice, SOD1^G93A^ mice are characterized by a disturbance of the global proteostasis, leading to a marked aggregation of p62 and ubiquitin [38]. To exclude that Atg9 accumulates due to an impaired proteostasis as a part of p62^+^ protein aggregates, we stained Atg9 along with p62. As previously described, we observed a marked accumulation of p62 in SOD1^G93A^ mice (Fig. 6a) [38]. In contrast, p62 accumulations were absent from the spinal cord cross-sections of *Plekhg5*-deficient mice (Fig. 6a). Notably, the Atg9^+^ vesicle clusters in SOD1^G93A^ mice did not stain positive for p62 or vice versa, suggesting that distinct mechanisms underlie these histopathological changes.

To examine whether physical exercise also leads to a reduction in the number of Atg9^+^ vesicles in SOD1^G93A^ mice, we subjected SOD1^G93A^ mice to four weeks of voluntary exercise and analyzed the number of Atg9^+^ clusters in the spinal cord sections (Fig. 6d, e). Compared to sedentary SOD1^G93A^ mice, we detected a marked reduction of Atg9^+^ clusters in all mice performing physical exercise (Fig. 6e). To follow up on our previous observation of increased sorting of SVs into the Atg9^+^ compartment, we analyzed the co-localization of SVs with Atg9^+^ clusters. Interestingly, SVs were rarely detectable in both sedentary and exercising mice (Fig. 6f, g). Furthermore, most Atg9^+^ clusters stained negative for the Lamp1 marker. Taken together, we conclude that the clustering of Atg9^+^ vesicles represents a broader disease mechanism in MND, which depends on neuronal activity. Furthermore, clearing of Atg9^+^ clusters by physical exercise is a valid approach to reducing the intracellular burden of accumulating vesicles and improving the histopathological disease progression.

## Discussion

Our data collectively show that physical exercise in young but not aged *Plekhg5*-deficient mice provides a tool to remove vesicle accumulations from axon terminals and preserve the NMJ integrity. While short-term exercise induced MN autophagy in young mice, it failed to trigger autophagy in aged animals, suggesting that the vesicle accumulations could not be cleared due to the age-dependent autophagy decline.

Atg9^+^ vesicles represent a unique but heterogeneous vesicle population that undergoes activity-dependent cycles of endo- and exocytosis in neurons [10, 11]. Our data demonstrate that depletion of Plekhg5 results in marked accumulation of Atg9^+^ vesicles in axon terminals. These vesicle accumulations are absent from MN somata and negative for the Golgi marker GM130. Atg9^+^ vesicles are normally processed via the endoplasmic reticulum and Golgi, followed by anterograde transport to presynaptic terminals, as previously shown in *C. elegans* [39]. Recent molecular profiling of Atg9-containing vesicles revealed that these vesicles are a heterogeneous population carrying distinct markers, including endo/lysosomal markers [11]. As the majority of Atg9^+^ clusters are positive for the late endosomal/lysosomal marker Lamp1, we conclude that the depletion of Plekhg5 leads to the accumulation of one of these vesicle populations that acquire Lamp1 during axonal transport or sorting at the presynaptic terminal [10, 11].

Previous work has suggested the cycling of Atg9^+^ vesicles between Atg9 reservoirs and pre-autophagosomal structures in presynaptic terminals during autophagosome biogenesis [10]. In line with our earlier work demonstrating defective presynaptic autophagy in *Plekhg5*-deficient mice [16], it is tempting to speculate that these deficits in presynaptic autophagy cause an accumulation of Atg9^+^ vesicles in such an Atg9 reservoir. Upon induction of neuronal activity by physical exercise, the accumulated Atg9^+^ vesicles are recruited from their reservoir, reducing the Atg9^+^ accumulations in 3-month-old mice.

Physical exercise provides multiple benefits to overall health while increasing the firing pattern of MNs [9]. While short-term physical exercise had only minor effects on the Agt9^+^ clusters, long-term exercise in 3-month-old mice reduced the Agt9^+^ clusters, preserved NMJ integrity, and improved motor performance. In contrast, physical exercise did not improve the aforementioned parameters in aged mice. In line with these data, physical exercise boosted MN autophagy in young animals but failed to trigger a similar effect in aged mice. To our surprise, physical exercise also induced MN autophagy in *Plekhg5*-deficient mice. These data demonstrate that Plekhg5-mediated SV turnover is a basal mechanism for maintaining the presynaptic compartment uncoupled from neuronal activity. Additionally, a second activity-dependent mechanism enables matching the autophagic flux to the physiological demands of neurons. This activity-dependent pathway provides a means to bypass the autophagy impairments in *Plekhg5*-deficient mice at a young age. Strikingly, the activity-dependent removal of the Atg9^+^ vesicles by presynaptic autophagy only occurred in young but not aged mice. This finding conceptually points to the age-dependent disconnection of neuronal activity and the required autophagy-mediated turnover. Our data indicate a central role of WIPI2B in bypassing the autophagy block in young *Plekhg5*-deficient mice by physical exercise. Stavoe et al. showed that WIPI2B counteracts the autophagy-dependent decline in neurons [33]. In agreement with the idea of dynamic WIPI2B phosphorylation to mediate membrane association and dissociation [33], we did not detect any striking differences in the membrane levels of WIPI2B in wild-type mice, regardless of age and physical exercise. In contrast, the phosphorylated form of WIPI2 accumulated in the membrane fraction of *Plekhg5*-deficient mice, suggesting an impaired membrane dissociation, possibly due to a defective dephosphorylation. It is tempting to speculate that physical exercise in young animals triggers a signaling cascade, which cumulates in the dephosphorylation of WIPI2B, facilitating its membrane association and dissociation to complete autophagosome biogenesis.

Previous studies showed an induction of autophagy with different forms of physical exercise [40, 41], even indicating beneficial effects in ALS [42]. However, to our knowledge, the interaction of autophagy induction via exercise and its link to the role of presynaptic homeostasis at an early stage in the pathophysiology of MND remains elusive. Furthermore, while the decline of autophagy with age has long been described [33, 43], the interaction between physical exercise-induced autophagy and age-dependent decline of MNs has not been described so far. Thus, our study has broad implications for neurodegenerative disorders where an age-dependent build-up of protein accumulations and aggregates is considered a central part of the pathophysiology [44, 45]. A limitation of our study is that while our data demonstrate that physical exercise for four weeks has beneficial effects on the histopathological level in young animals, we did not assess any potential long-lasting effects. Therefore, further investigation should focus on the potential long-lasting protective effects of exercise in an in vivo mouse model. Although continuous life-long spontaneous exercise does not improve lifespan, improvements in motor coordination and muscle strength have been previously reported [46, 47].

NMJs are among the earliest targets in the pathophysiological cascade of MND. Therefore, it is important to understand the mechanisms that contribute to the maintenance of the presynaptic compartment [48]. To explore whether the accumulation of Atg9^+^ vesicles represents a general hallmark of presynaptic dysfunction in MND, we analyzed the distribution of Atg9 in spinal cord sections of SOD1^G93A^ mice. Notably, we also found Atg9^+^ accumulations in these mice, suggesting a broader disease relevance beyond *PLEKHG5* variant-related MNDs. Although the underlying mechanism leading to the clustering of Atg9^+^ vesicles may differ between *Plekhg5*-deficient and SOD1^G93A^ mice, both vesicle populations can be removed by physical exercise until a certain age. Whereas the majority of Atg9^+^ vesicles in *Plekhg*5-deficient mice appeared positive for Lamp1, Lamp1 was absent from most Atg9^+^ vesicle clusters in SOD1^G93A^ mice. The recently described heterogeneity of neuronal Atg9^+^ vesicles might provide a potential explanation for the difference in the membrane protein composition between Atg9^+^ vesicle clusters [11]. In both models, the Atg9^+^ accumulations did not overlap with p62^+^ clusters, showing that protein aggregation occurs independently of vesicle accumulation in SOD1^G93A^ mice. Notably, Atg9^+^ clusters were similarly removed upon physical exercise in *Plekhg5*-deficient mice, strongly emphasizing the validity of this approach in improving the pathophysiology of different MNDs. Notably, a recent study identified a pathophysiological link between SOD1 and Plekhg5, demonstrating that Plekhg5 regulates the secretory autophagy of SOD1 [29]. Our findings here support the notion that Plekhg5 contributes to the pathophysiology of SOD1-ALS and possibly also other forms of MND.

Our work extends the concept that presynaptic dysfunction is an early event in the pathophysiology of MND characterized by Atg9^+^ vesicle accumulations. An early interference with vesicle clustering by physical exercise appears as a promising strategy to preserve motor function and disease progression throughout different MNDs.

## Conclusion

Our study highlights that presynaptic dysfunction is an early event in the pathophysiology of MND characterized by Atg9^+^ vesicle accumulations. Voluntary running wheel exercise in young but surprisingly not in aged *Plekhg5*-deficient mice triggered the removal of Atg9^+^ vesicle accumulations and improved NMJ integrity. In line with an age-dependent decline of neuronal autophagy, short-term voluntary exercise triggered MN autophagy in young but not old mice. Conceptually, our findings point to the age-dependent disconnection of neuronal activity and the required autophagy-mediated turnover that might explain the age-dependent build-up of protein accumulations and aggregates as the central part of the pathophysiology in neurodegenerative disorders. Pointing to a broader role of Atg9-containing vesicles in the pathophysiology of MND, we also detected Atg9-containing vesicle accumulations in SOD1^G93A^ mice. Strikingly, physical exercise in presymptomatic SOD1^G93A^ mice resulted in the reduction of the vesicle accumulations. We conclude that early presymptomatic interference with vesicle clustering by physical exercise appears to be a promising strategy for preserving motor function and disease progression.

## Supplementary Information

 Additional file 1. **Table S1**. Mouse strains. **Table S2**. Primary antibodies for immunohistochemistry.  **Table S3**. Secondary antibodies for immunohistochemistry. **Table S4**. Primary antibodies for western blot. **Table S5**. Home-made set up for STORM and fluorescence images. **Table S6**. Image process details and resolution from Epifluorescence and SMLM images. **Fig S1**. Four hours of voluntary exercise is insufficient to clear Atg9^+^ clusters. **Fig S2**. Further analysis of Atg9^+^ clusters and NMJs upon physical exercise. **Fig S3**. Atg9^+^ clusters are comprised of individual vesicles, whereas Atg9 and Lamp1 are present on the same vesicular membrane. **Fig S4**. The autophagic process is spatially separated in MNs and inducible by neuronal activity in vitro.

Additional file 2. Uncropped western blots.

## Data Availability

The data supporting the findings of this study are available from the corresponding authors upon reasonable request.

## Abbreviations

AChR: Acetylcholine receptor
ALS: Amyotrophic lateral sclerosis
ChAT: Choli acetyl transferase
CNX: Calnexin
dSTORM: Direct stochastic optical reconstruction microscopy
GAS: Gastrocnemius
MN: Motoneuron
MND: Motoneuron disease
NMJ: Neuromuscular junction
p-WIPI2: Phosphorylated WIPI2
SV: Synaptic vesicles
TA: Tibialis anterior

## References

[1] Komatsu M, Waguri S, Chiba T, Murata S, Iwata J, Tanida I, et al. Loss of autophagy in the central nervous system causes neurodegeneration in mice. Nature. 2006;441(7095):880–4. 16625205 10.1038/nature04723

[2] Hara T, Nakamura K, Matsui M, Yamamoto A, Nakahara Y, Suzuki-Migishima R, et al. Suppression of basal autophagy in neural cells causes neurodegenerative disease in mice. Nature. 2006;441(7095):885–9. 16625204 10.1038/nature04724

[3] Luningschror P, Sendtner M. Autophagy in the presynaptic compartment. Curr Opin Neurobiol. 2018;51:80–5. 29549710 10.1016/j.conb.2018.02.023

[4] Vijayan V, Verstreken P. Autophagy in the presynaptic compartment in health and disease. J Cell Biol. 2017;216(7):1895–906. 28515275 10.1083/jcb.201611113 PMC5496617

[5] Hill SE, Colon-Ramos DA. The journey of the synaptic autophagosome: a cell biological perspective. Neuron. 2020;105(6):961–73. 32191859 10.1016/j.neuron.2020.01.018

[6] Decet M, Verstreken P. Presynaptic autophagy and the connection with neurotransmission. Front Cell Dev Biol. 2021;9:790721. 34988081 10.3389/fcell.2021.790721 PMC8722708

[7] Chanaday NL, Cousin MA, Milosevic I, Watanabe S, Morgan JR. The synaptic vesicle cycle revisited: new insights into the modes and mechanisms. J Neurosci. 2019;39(42):8209–16. 31619489 10.1523/JNEUROSCI.1158-19.2019 PMC6794917

[8] Jahne S, Mikulasch F, Heuer HGH, Truckenbrodt S, Agui-Gonzalez P, Grewe K, et al. Presynaptic activity and protein turnover are correlated at the single-synapse level. Cell Rep. 2021;34(11):108841. 33730575 10.1016/j.celrep.2021.108841

[9] Gardiner P, Dai Y, Heckman CJ. Effects of exercise training on alpha-motoneurons. J Appl Physiol (1985). 2006;101(4):1228–36. 16778002 10.1152/japplphysiol.00482.2006

[10] Yang S, Park D, Manning L, Hill SE, Cao M, Xuan Z, et al. Presynaptic autophagy is coupled to the synaptic vesicle cycle via ATG-9. Neuron. 2022;110(5):824–40. 35065714 10.1016/j.neuron.2021.12.031 PMC9017068

[11] Binotti B, Ninov M, Cepeda AP, Ganzella M, Matti U, Riedel D, et al. ATG9 resides on a unique population of small vesicles in presynaptic nerve terminals. Autophagy. 2024;20(4):883–901. 37881948 10.1080/15548627.2023.2274204 PMC11062364

[12] Matoba K, Kotani T, Tsutsumi A, Tsuji T, Mori T, Noshiro D, et al. Atg9 is a lipid scramblase that mediates autophagosomal membrane expansion. Nat Struct Mol Biol. 2020;27(12):1185–93. 33106658 10.1038/s41594-020-00518-w

[13] Kishi-Itakura C, Koyama-Honda I, Itakura E, Mizushima N. Ultrastructural analysis of autophagosome organization using mammalian autophagy-deficient cells. J Cell Sci. 2014;127(18):4089–102. 25052093 10.1242/jcs.156034

[14] Yamaguchi J, Suzuki C, Nanao T, Kakuta S, Ozawa K, Tanida I, et al. Atg9a deficiency causes axon-specific lesions including neuronal circuit dysgenesis. Autophagy. 2018;14(5):764–77. 28513333 10.1080/15548627.2017.1314897 PMC6070006

[15] Binotti B, Pavlos NJ, Riedel D, Wenzel D, Vorbruggen G, Schalk AM, et al. The GTPase Rab26 links synaptic vesicles to the autophagy pathway. Elife. 2015;4:e05597. 25643395 10.7554/eLife.05597 PMC4337689

[16] Luningschror P, Binotti B, Dombert B, Heimann P, Perez-Lara A, Slotta C, et al. Plekhg5-regulated autophagy of synaptic vesicles reveals a pathogenic mechanism in motoneuron disease. Nat Commun. 2017;8(1):678. 29084947 10.1038/s41467-017-00689-z PMC5662736

[17] Senderek J. PLEKHG5: merging phenotypes and disease mechanisms in Charcot-Marie-Tooth neuropathy and lower motor neuron disease. Eur J Neurol. 2021;28(4):1106–7. 33492783 10.1111/ene.14752

[18] Maystadt I, Rezsohazy R, Barkats M, Duque S, Vannuffel P, Remacle S, et al. The nuclear factor kappaB-activator gene PLEKHG5 is mutated in a form of autosomal recessive lower motor neuron disease with childhood onset. Am J Hum Genet. 2007;81(1):67–76. 17564964 10.1086/518900 PMC1950913

[19] Chen Z, Maroofian R, Basak AN, Shingavi L, Karakaya M, Efthymiou S, et al. Novel variants broaden the phenotypic spectrum of PLEKHG5-associated neuropathies. Eur J Neurol. 2021;28(4):1344–55. 33220101 10.1111/ene.14649

[20] Luningschror P, Slotta C, Heimann P, Briese M, Weikert UM, Massih B, et al. Absence of Plekhg5 results in myelin infoldings corresponding to an impaired schwann cell autophagy and a reduced T-cell infiltration into peripheral nerves. Front Cell Neurosci. 2020;14:185. 32733205 10.3389/fncel.2020.00185 PMC7358705

[21] Griebel M, Segebarth D, Stein N, Schukraft N, Tovote P, Blum R, et al. Deep learning-enabled segmentation of ambiguous bioimages with deepflash2. Nat Commun. 2023;14(1):1679. 36973256 10.1038/s41467-023-36960-9 PMC10043282

[22] Wirths O. Preparation of crude synaptosomal fractions from mouse brains and spinal cords. Bio Protoc. 2017;7(15):e2423. 34541151 10.21769/BioProtoc.2423 PMC8413490

[23] Forssmann WG, Ito S, Weihe E, Aoki A, Dym M, Fawcett DW. An improved perfusion fixation method for the testis. Anat Rec. 1977;188(3):307–14. 332010 10.1002/ar.1091880304

[24] Welsch MMU. Romeis—Mikroskopische technik. Springer; 2015.

[25] Appeltshauser L, Linke J, Heil HS, Karus C, Schenk J, Hemmen K, et al. Super-resolution imaging pinpoints the periodic ultrastructure at the human node of Ranvier and its disruption in patients with polyneuropathy. Neurobiol Dis. 2023;182:106139. 37146836 10.1016/j.nbd.2023.106139

[26] Ovesny M, Krizek P, Borkovec J, Svindrych Z, Hagen GM. ThunderSTORM: a comprehensive ImageJ plug-in for PALM and STORM data analysis and super-resolution imaging. Bioinfor. 2014;30(16):2389–90. 10.1093/bioinformatics/btu202 PMC4207427 24771516

[27] Abramoff MDM, Paulo J, Ram SJ. Image processing with ImageJ. Biophotonics Int. 2024;11(7):36–42.

[28] Wiese S, Herrmann T, Drepper C, Jablonka S, Funk N, Klausmeyer A, et al. Isolation and enrichment of embryonic mouse motoneurons from the lumbar spinal cord of individual mouse embryos. Nat Protoc. 2010;5(1):31–8. 20057379 10.1038/nprot.2009.193

[29] Hutchings AJ, Hambrecht B, Veh A, Giridhar NJ, Zare A, Angerer C, et al. Plekhg5 controls the unconventional secretion of Sod1 by presynaptic secretory autophagy. Nat Commun. 2024;15(1):8622. 39366938 10.1038/s41467-024-52875-5 PMC11452647

[30] De Pace R, Skirzewski M, Damme M, Mattera R, Mercurio J, Foster AM, et al. Altered distribution of ATG9A and accumulation of axonal aggregates in neurons from a mouse model of AP-4 deficiency syndrome. PLoS Genet. 2018;14(4):e1007363. 29698489 10.1371/journal.pgen.1007363 PMC5940238

[31] Giacomello E, Crea E, Torelli L, Bergamo A, Reggiani C, Sava G, et al. Age dependent modification of the metabolic profile of the tibialis anterior muscle fibers in C57BL/6J mice. Int J Mol Sci. 2020. 10.3390/ijms21113923 PMC7312486 32486238

[32] Manttari S, Jarvilehto M. Comparative analysis of mouse skeletal muscle fibre type composition and contractile responses to calcium channel blocker. BMC Physiol. 2005;5(1):4. 15710036 10.1186/1472-6793-5-4 PMC550649

[33] Stavoe AK, Gopal PP, Gubas A, Tooze SA, Holzbaur EL. Expression of WIPI2B counteracts age-related decline in autophagosome biogenesis in neurons. Elife. 2019. 10.7554/eLife.44219 PMC6634969 31309927

[34] Kimura S, Noda T, Yoshimori T. Dissection of the autophagosome maturation process by a novel reporter protein tandem fluorescent-tagged LC3. Autophagy. 2007;3(5):452–60. 17534139 10.4161/auto.4451

[35] Ariosa AR, Klionsky DJ. Autophagy core machinery: overcoming spatial barriers in neurons. J Mol Med (Berl). 2016;94(11):1217–27. 27544281 10.1007/s00109-016-1461-9 PMC5071157

[36] Lee JH, Yang DS, Goulbourne CN, Im E, Stavrides P, Pensalfini A, et al. Faulty autolysosome acidification in Alzheimer’s disease mouse models induces autophagic build-up of Abeta in neurons yielding senile plaques. Nat Neurosci. 2022;25(6):688–701. 35654956 10.1038/s41593-022-01084-8 PMC9174056

[37] Maday S, Wallace KE, Holzbaur EL. Autophagosomes initiate distally and mature during transport toward the cell soma in primary neurons. J Cell Biol. 2012;196(4):407–17. 22331844 10.1083/jcb.201106120 PMC3283992

[38] Brenner D, Sieverding K, Bruno C, Luningschror P, Buck E, Mungwa S, et al. Heterozygous Tbk1 loss has opposing effects in early and late stages of ALS in mice. J Exp Med. 2019;216(2):267–78. 30635357 10.1084/jem.20180729 PMC6363427

[39] Stavoe AK, Hill SE, Hall DH, Colon-Ramos DA. KIF1A/UNC-104 transports ATG-9 to regulate neurodevelopment and autophagy at synapses. Dev Cell. 2016;38(2):171–85. 27396362 10.1016/j.devcel.2016.06.012 PMC4961624

[40] He C, Sumpter R Jr., Levine B. Exercise induces autophagy in peripheral tissues and in the brain. Autophagy. 2012;8(10):1548–51. 22892563 10.4161/auto.21327 PMC3463459

[41] Jamart C, Francaux M, Millet GY, Deldicque L, Frere D, Feasson L. Modulation of autophagy and ubiquitin-proteasome pathways during ultra-endurance running. J Appl Physiol (1985). 2012;112(9):1529–37. 22345427 10.1152/japplphysiol.00952.2011

[42] Desseille C, Deforges S, Biondi O, Houdebine L, D’Amico D, Lamaziere A, et al. Specific physical exercise improves energetic metabolism in the skeletal muscle of amyotrophic-lateral- sclerosis mice. Front Mol Neurosci. 2017;10:332. 29104532 10.3389/fnmol.2017.00332 PMC5655117

[43] Khalil H, Tazi M, Caution K, Ahmed A, Kanneganti A, Assani K, et al. Aging is associated with hypermethylation of autophagy genes in macrophages. Epigenetics. 2016;11(5):381–8. 26909551 10.1080/15592294.2016.1144007 PMC4889231

[44] Palmer JE, Wilson N, Son SM, Obrocki P, Wrobel L, Rob M, et al. Autophagy aging and age-related neurodegeneration. Neuron. 2025;113(1):29–48. 39406236 10.1016/j.neuron.2024.09.015

[45] Simonsen A, Wollert T. Don’t forget to be picky—selective autophagy of protein aggregates in neurodegenerative diseases. Curr Opin Cell Biol. 2022;75:102064. 35240373 10.1016/j.ceb.2022.01.009

[46] Garcia-Valles R, Gomez-Cabrera MC, Rodriguez-Manas L, Garcia-Garcia FJ, Diaz A, Noguera I, et al. Life-long spontaneous exercise does not prolong lifespan but improves health span in mice. Longev Healthspan. 2013;2(1):14. 24472376 10.1186/2046-2395-2-14 PMC3922914

[47] Feng M, Li M, Lou J, Wu G, Gao T, Wu F, et al. Early-life exercise extends healthspan but not lifespan in mice. Nat Commun. 2025;16(1):6328. 40634291 10.1038/s41467-025-61443-4 PMC12241455

[48] Murray LM, Talbot K, Gillingwater TH. Review: neuromuscular synaptic vulnerability in motor neurone disease: amyotrophic lateral sclerosis and spinal muscular atrophy. Neuropathol Appl Neurobiol. 2010;36(2):133–56. 20202121 10.1111/j.1365-2990.2010.01061.x

