# Supplementary material for: Age-dependent removal of Atg9-containing vesicle accumulations in motoneuron disease models by physical exercise
Source: Transl Neurodegener. 2025 Dec 16;14:69. doi: 10.1186/s40035-025-00524-2 (PMC12706973; doi:10.1186/s40035-025-00524-2)
Supplement: Supplementary file 1 — Additional file 1. Table S1. Mouse strains. Table S2. Primary antibodies for immunohistochemistry. Table S3. Secondary antibodies for immunohistochemistry. Table S4. Primary antibodies for western blot. Table S5. Home-made set up for STORM and fluorescence images. Table S6. Image process details and resolution from Epifluorescence and SMLM images. Fig S1. Four hours of voluntary exercise is insufficient to clear Atg9+ clusters. Fig S2. Further analysis of Atg9+ clusters and NMJs upon physical exercise. Fig S3. Atg9+ clusters are comprised of individual vesicles, whereas Atg9 and Lamp1 are present on the same vesicular membrane. Fig S4. The autophagic process is spatially separated in MNs and inducible by neuronal activity in vitro. [file 40035_2025_524_MOESM1_ESM.docx]

**Table S1. Mouse strains**

| Name | Genetic background | Source |
| --- | --- | --- |
| Plekhg5^-/-^ | B6.Pekgh5/J | (1) |
| Thy1::YFP | B6.Cg-Tg(Thy1-YFPH)23Jrs/J | Jackson Lab, #003782 |
| mRFP-GFP-LC3 | C57BL/6-Tg(CAG-RFP/EGFP/Map1lc3b)1Hill/J | Jackson Lab, #027139 |
| SOD1^G93A^ | B6SJL-TgN(SOD1-G93A)^dl^1Gur/J | Jackson Lab, #002300 |

**Table S2. Primary antibodies for immunohistochemistry**

| Name | Company | City, Country | Catalogue number |
| --- | --- | --- | --- |
| Atg9 | Abcam | Cambridge, UK | Ab108338 |
| Lamp1 | Invitrogen | Dreieich, Germany | 14-1071-82 |
| Synaptophysin | Synaptic systems | Göttingen, Germany | 101 004 |
| NFH | Millipore Sigma | Darmstadt, Germany | ab5539 |
| GFP | Abcam | Cambridge, UK | ab13970 |
| RFP | Rockland | Aachen, Germany | 600-401-379 |
| RFP | SicGen antibodies | Berlin, Germany | AB1140-100 |
| GM130 | BD Biosciences | Heidelberg, Germany | 610822 |
| ChAT | Millipore Sigma | Darmstadt, Germany | ab144P |
| p62 | Progen | Heidelberg, Germany | GP62-C |

**Table S3. Secondary antibodies for immunohistochemistry**

| Name | Company | City, Country | Catalogue number |
| --- | --- | --- | --- |
| Anti-rabbit Alexa488 | Jackson ImmunoResearch | Cambridgeshire, UK | 711-545-152 |
| Anti-rabbit Cy3 | Jackson ImmunoResearch | Cambridgeshire, UK | 711-165-152 |
| Anti-rabbit Cy5 | Jackson ImmunoResearch | Cambridgeshire, UK | 711-175-152 |
| Anti-mouse Alexa488 | Jackson ImmunoResearch | Cambridgeshire, UK | 715-545-151 |
| Anti-chicken Alexa488 | Jackson ImmunoResearch | Cambridgeshire, UK | 703-545-155 |
| Anti-chicken Cy3 | Jackson ImmunoResearch | Cambridgeshire, UK | 703-165-155 |
| Anti-rat Alexa488 | Jackson ImmunoResearch | Cambridgeshire, UK | 712-545-150 |
| Anti-rat Cy3 | Jackson ImmunoResearch | Cambridgeshire, UK | 712-165-150 |
| Anti-guinea pig Alexa488 | Jackson ImmunoResearch | Cambridgeshire, UK | 706-545-148 |
| Anti-guinea pig Cy5 | Jackson ImmunoResearch | Cambridgeshire, UK | 706-175-148 |
| Anti-goat Alexa488 | Jackson ImmunoResearch | Cambridgeshire, UK | 705-545-003 |

**Table S4. Primary antibodies for western blot**

| Name | Company | City, Country | Catalogue number |
| --- | --- | --- | --- |
| Atg9 | Abcam | Cambridge, UK | Ab108338 |
| Lamp1 | Invitrogen | Dreieich, Germany | 14-1071-82 |
| Calnexin | SicGen antibodies | Berlin, Germany | AB0041-200 |
| LC3B | Novus Biologicals | Wiesbaden-Nordenstadt, Germany | NB100-2220 |
| GAPDH | Millipore Sigma | Darmstadt, Germany | CB1001 |
| WIPI2 | Abcam | Cambridge, UK | ab105459 |
| p-WIPI2 | Cell signaling | Darmstadt, Germany | 13571S |

**Table S5. Home-made set up for STORM and fluorescence images**

| Illumination | 640 nm laser (iBEAM-smart-640-S, Toptica), 532 nm laser (GEM 532 nm, 250 mW, Laser Qantum) |
| --- | --- |
| Clean up filters | 640/8 nm MaxDiode^TM^ laser clean-up, Semrock Filter ZET 532/10 clean-up, Chroma |
| Dichroic filter | QuadLine Laser-Beamsplitter R405/488/532/635, Semrock Quad-Notch Filter 405/488/532/635, Semrock |
| Emission filters | BrightLine HC 593/40, Semrock 700/75 ET, Chroma |
| Camera | iXon ultra DU-897U.CSO- #BV, Andor |

**Table S6. Image process details and resolution from Epifluorescence and SMLM images**

| Zeiss Observer Z.1, Carl Zeiss AG | | |
| --- | --- | --- |
|  | Epifluorescence | SMLM |
| Objectives | Alpha Plan-Apochromat 63k NA 1.46 Oil Korr, 420780-9970 | Alpha Plan-Apochromat 63k NA 1.46 Oil Korr, 420780-9970 |
| Detector/camera | EMCCD Camera EM-Gain: 100, 17.7 photoelectrons per A/D count | EMCCD Camera EM-Gain: 100, 17.7 photoelectrons per A/D count |
| Pixels per FoV | 340*340 | 170*170 |
| Voxels/pixel size | x,y = 204 nm | x,y = 102 nm |
| Channels | 532 nm, 639 nm 0-1 kW cm^2^ | 532 nm, 639 nm 1-5 kW cm^2^ |
| Acquisition | 100 fr (20 ms (Ch1)/20 ms (Ch2)) exposure time | 20000 fr (20 ms (Ch1)/20 ms (Ch2)) exposure time |
| Image processing | Image stack – Average intensity | ThundersSTORM: Image filtering: Waveletfilter (B-spline), order: 3, scale 2.0;  Approximate localization of molecules: Local maximum, peak intensity threshold: x*std(Wave.F1) (x=1.25-6), 8-neighborhood.  Sub-pixel localization of molecules: Integrated gaussian PSF, fitting radius [px]: 3, weighted least squares, initial sigma [px]: 1.6.  Drift correction was performed with autocorrelation. Filter: Sigma <130-190. |

**References**

1. Luningschror P, Binotti B, Dombert B, Heimann P, Perez-Lara A, Slotta C, et al. Plekhg5-regulated autophagy of synaptic vesicles reveals a pathogenic mechanism in motoneuron disease. Nat Commun. 2017;8(1):678.


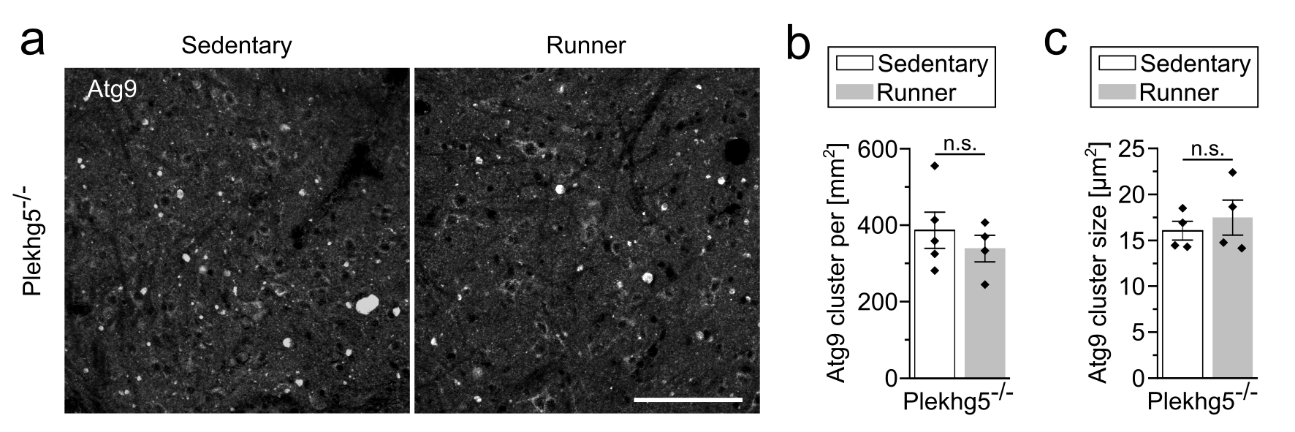


**Fig. S1 Four hours of voluntary exercise is insufficient to clear Atg9^+^ clusters.**

**a** Tendency towards reduced Atg9^+^ clusters with four hours of voluntary physical exercise. Lumbar spinal cord cross sections from wildtype and *Plekhg5*-deficient mice with immunohistochemical labeling of Atg9. Scale bar 50µm. **b** Quantification of Atg9^+^ clusters in lumbar spinal cord tissue from *Plekhg5*-deficient mice after four hours of voluntary physical exercise. Each data point represents the mean from seven spinal cord sections per mouse. Sedentary *n*=4; Runner *n*=4. Two sample T-test. **c** Quantification of Atg9 cluster size in lumbar spinal cord sections from *Plekhg5*-deficient mice. Each data point represents the mean size of all Atg9^+^ clusters from seven spinal cord sections from each mouse. Sedentary *n*=4; Runner *n*=4. Two sample T-test. All data are shown as ± SEM; n.s., not significant (*P* > 0.05); all representative images are taken from at least 3 biological replicates.


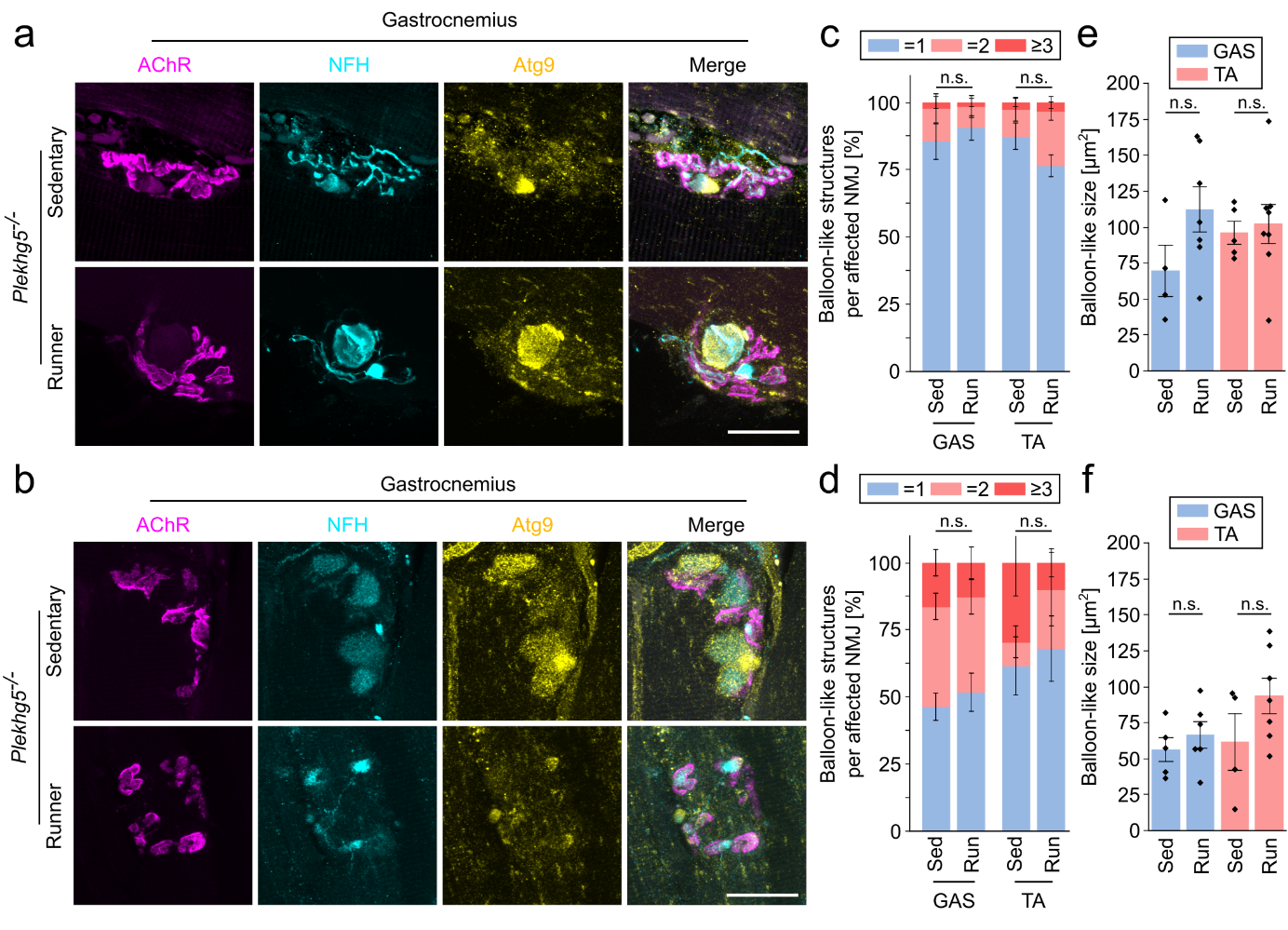


**Fig. S2 Further analysis of Atg9^+^ clusters and NMJs upon physical exercise.**

**a, b** Atg9 accumulates in balloon-like structures in NMJs from GAS muscles in 3-month-old (**a**) and 12-month-old (**b**) *Plekhg5*-deficient mice regardless of exercise. Immunofluorescence of NFH and Atg9 with postsynaptic staining via BTX conjugated fluorophore in 3-month-old *Plekhg5*-deficient mice. Scale bar 25µm. **c, d** Number of balloon-like clusters per affected NMJ in GAS and TA muscles from 3-month-old (**c**) and 12-month-old (**d**) *Plekhg5*-deficient mice. Values are given as percentage from all balloon-like clusters. Bars represent mean value from individual mice consisting of 15 - 41 NMJs each. A: GAS-Sed, *n*=4; GAS-Run, *n*=7; TA-Sed, *n*=5; TA-Run, *n*=8. B: GAS-Sed, *n*=5; GAS-Run, *n*=6; TA-Sed, *n*=4; TA-Run, *n*=7. Two-way ANOVA; Holm-Bonferroni multiple comparison test. **e, f** Size of balloon-like clusters in 3-month-old (**e**) and 12-month-old (**f**) *Plekhg5*-deficient mice from GAS and TA muscles. Each dot represents the mean value from one mouse with 15 - 41 NMJs analyzed each. (**e**) GAS-Sed, *n*=4; GAS-Run, *n*=7; TA-Sed, *n*=5; TA-Run, *n*=8. (**f**) GAS-Sed, *n*=5; GAS-Run, *n*=6; TA-Sed, *n*=4; TA-Run, *n*=7. Two-way ANOVA; Holm-Bonferroni multiple comparison test. All data are shown as ± SEM; n.s., not significant (*P* > 0.05); all representative images are taken from at least 3 biological replicates.


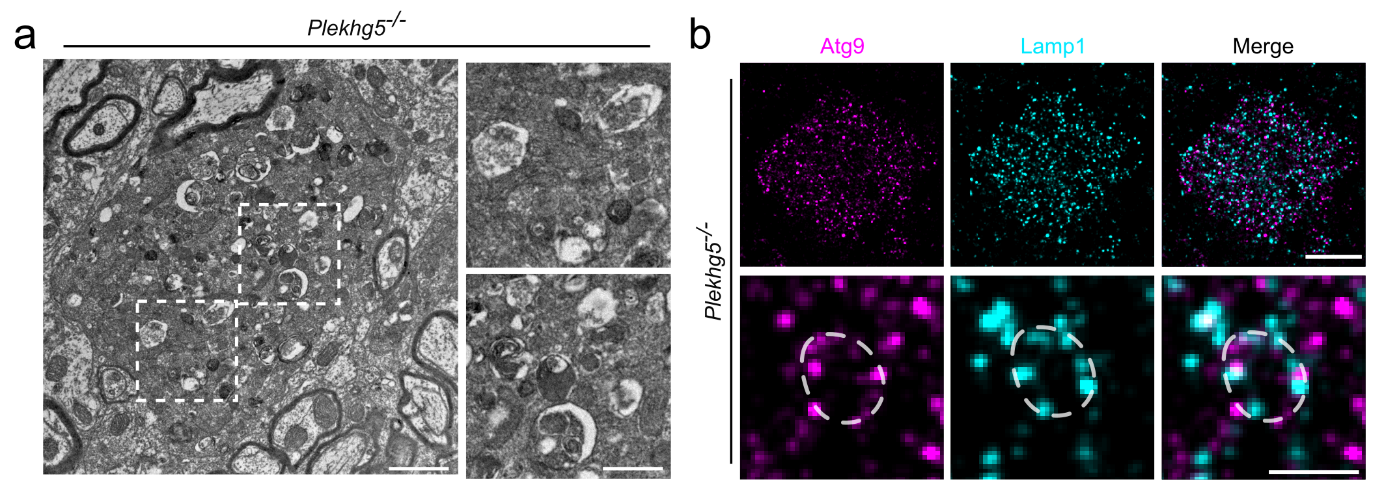


**Fig. S3 Atg9^+^ clusters are comprised of individual vesicles, whereas Atg9 and Lamp1 are present on the same vesicular membrane. a** Vesicle clusters are comprised of heterogeneous, individual, densely packed single-membrane vesicles. Electron microscopic image of lumbar spinal cord cross sections from *Plekhg5*-deficient mice. Scale bar left panel 1 µm, scale bar right panels 500 nm. **b** Atg9^+^ and Lamp1^+^ puncta are present on the same vesicle membrane, identifiable by a circular pattern. Immunofluorescence of Atg9 and Lamp1 imaged via super resolution microscopy utilizing dSTORM. Scale bar upper panel 2 µm, scale bar lower panel 500 nm. All figures are taken from at least 3 biological replicates. EM images are taken from at least 3 technical replicates.


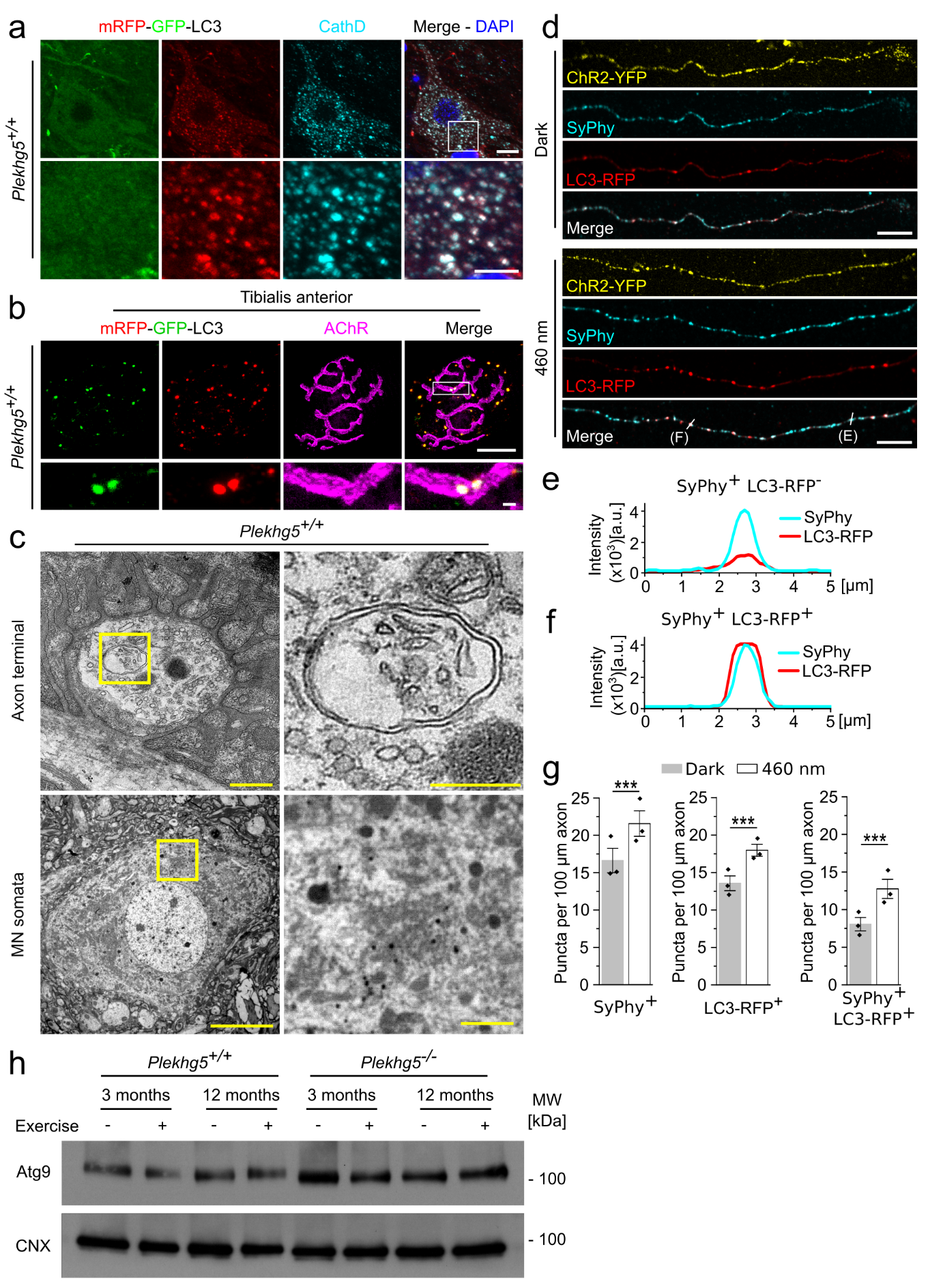


**Fig. S4 The autophagic process is spatially separated in MNs and inducible by neuronal activity in vitro. a** RFP^+^ puncta within the MN soma represent fused autolysosomes. MN soma within spinal cord sections from mRFP-GFP-LC3 transgenic mice. Immunohistochemical staining of the lysosomal marker CathD and amplified signal of GFP and mRFP via antibodies. Nucleus visualized via DAPI. GFP staining is omitted in the merge panel. Scale bar upper panel 10 µm; Scale bar lower panel inset 5µm. **b** GFP^+^ RFP^+^ double-positive puncta represent autophagosomes in the NMJs from tibialis anterior muscles. Double-positive GFP^+^ and RFP^+^ structures are visible in NMJs of 3-month-old wild-type and *Plekhg5*-deficient mice. Mice were crossed with the LC3-mRFP-GFP mice to visualize LC3. Immunofluorescence of GFP and RFP linked to LC3 with the postsynapse labeled by BTX. Scale bar 30 µm, scale bar blow-up 2 µm. **c** Spatial segmentation of autophagosomes is evident in MN somata and NMJs. Ultrastructure of NMJ and MN in lumbar spinal cord sections. Double-membraned autophagosomes are visible at axon terminals and absent from MN soma. Electron-dense lysosomes are present only within somata. Scale bar upper left panel 500 nm, scale bar upper right panel 250 nm, scale bar lower left panel 20 µm, scale bar lower right panel 2 µm. **d** Induction of neuronal activity by blue light increases the number of autophagosomes and synaptic vesicles. LC3-RFP and YFP-ChR2 expressing primary MNs with either unstimulated or blue light stimulated condition at 460 nm. ChR2-GFP expression in yellow, synaptophysin in cyan, LC3-RFP in red, and merge in white. Scale bar 10µm.

**e, f** Line scan of two Synaptophysin^+^ puncta within the axon. Single Synaptophysin^+^ puncta in (**e**) and Synaptophysin^+^ LC3-RFP^+^ double-positive puncta in (**f**). **g** Quantification of Synaptophysin^+^, LC3-RFP^+^, and Synaptophysin^+^ LC3-RFP^+^ double-positive puncta per 100 µm axon. Each data point represents one biological replicate with the mean value from 15 individual images. Dark *n*=3; 460nm *n*=3. Paired sample T-test; ± SEM; ****P* < 0.001. **h** Western Blot showing reduced levels of Atg9 after physical exercise in 3-month-old mice. The Atg9 levels were normalized to calnexin, and the value of 3-month-old wildtype sedentary mice was set to 1. Spinal cord homogenates from at least three mice were pooled to obtain the membrane pellets. All representative images are taken from at least 3 biological replicates. EM images are taken from at least 3 technical replicates.
