# Supplementary material for: Age-dependent removal of Atg9-containing vesicle accumulations in motoneuron disease models by physical exercise
Source: Transl Neurodegener. 2025 Dec 16;14:69. doi: 10.1186/s40035-025-00524-2 (PMC12706973; doi:10.1186/s40035-025-00524-2)
Supplement: Supplementary file 2 — Additional file 2. Uncropped western blots. [file 40035_2025_524_MOESM2_ESM.docx]

**Supplementary data: Uncropped western blots**

Uncropped western blots with corresponding bands appearing in the manuscript and supplementary figures marked by red boxes.

Fig.5 F: LC3

**
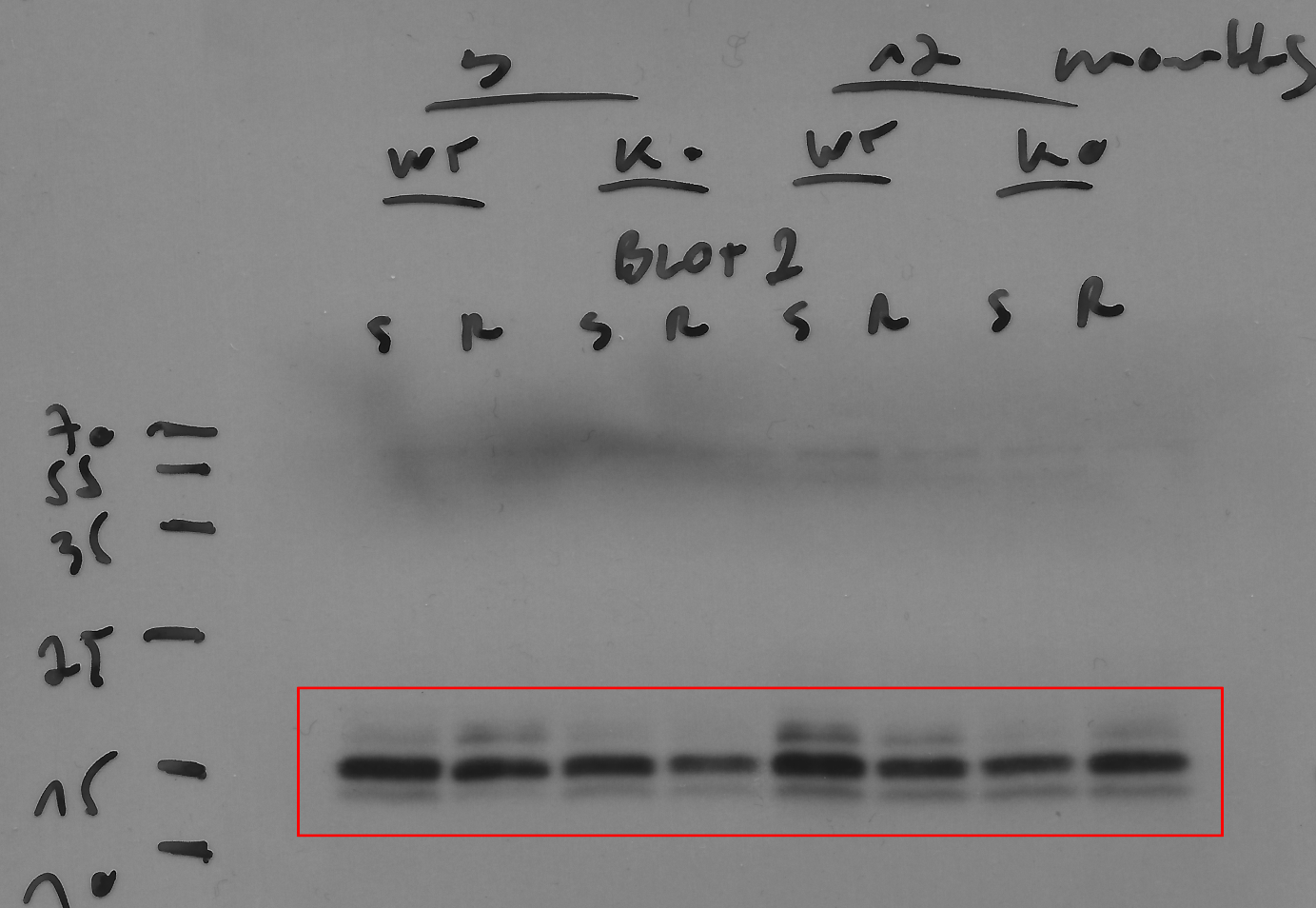
**

Fig.5 F: Gapdh

**
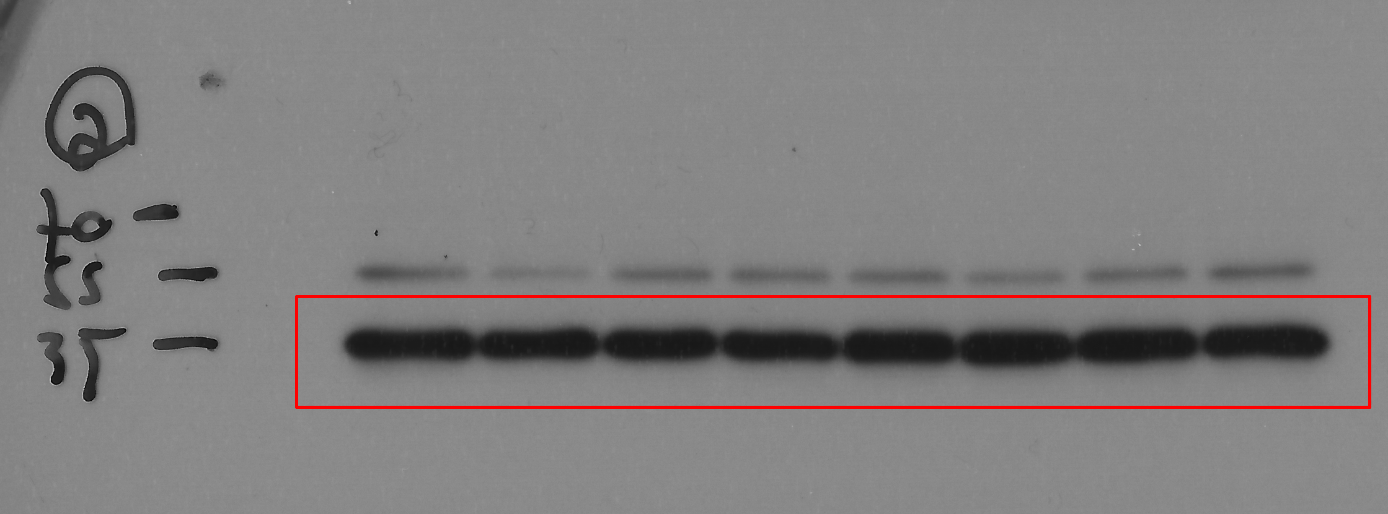
**

Fig.5 H: Atg9

**
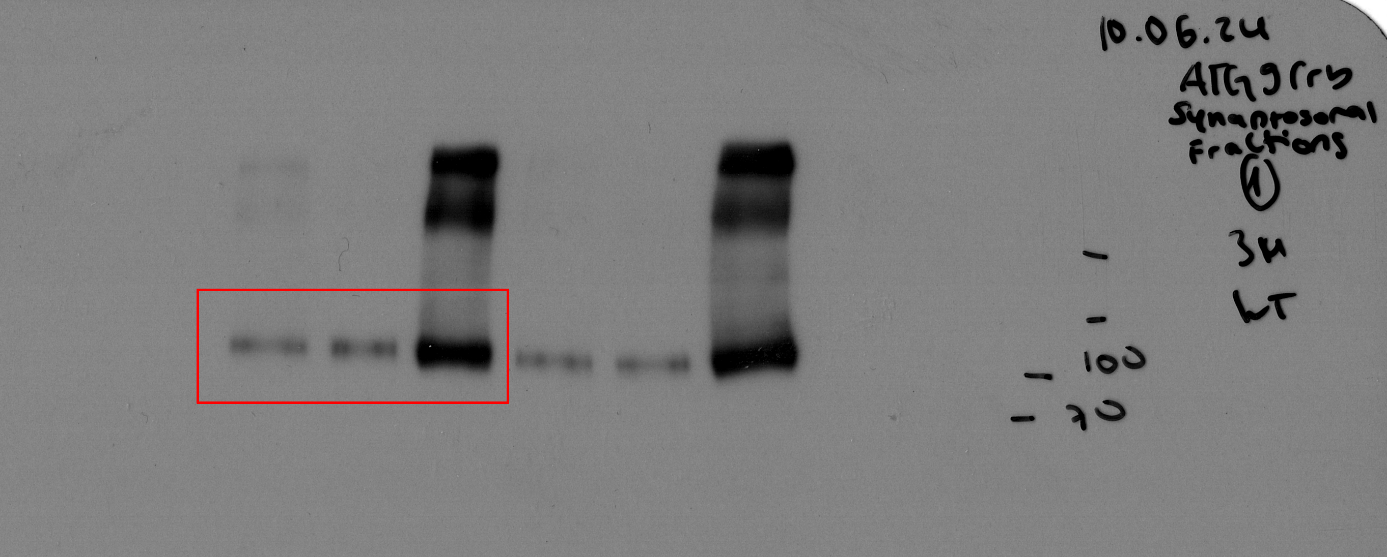
**

Fig.5 H: Lamp1

**
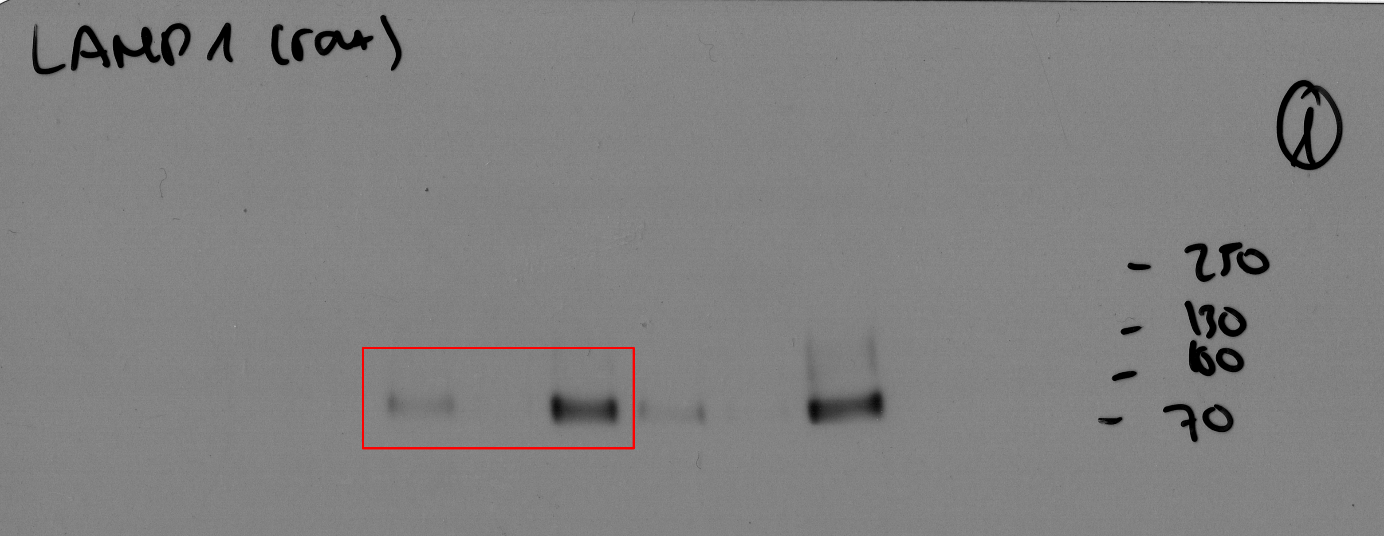
**

Fig.5 H: CNX

**
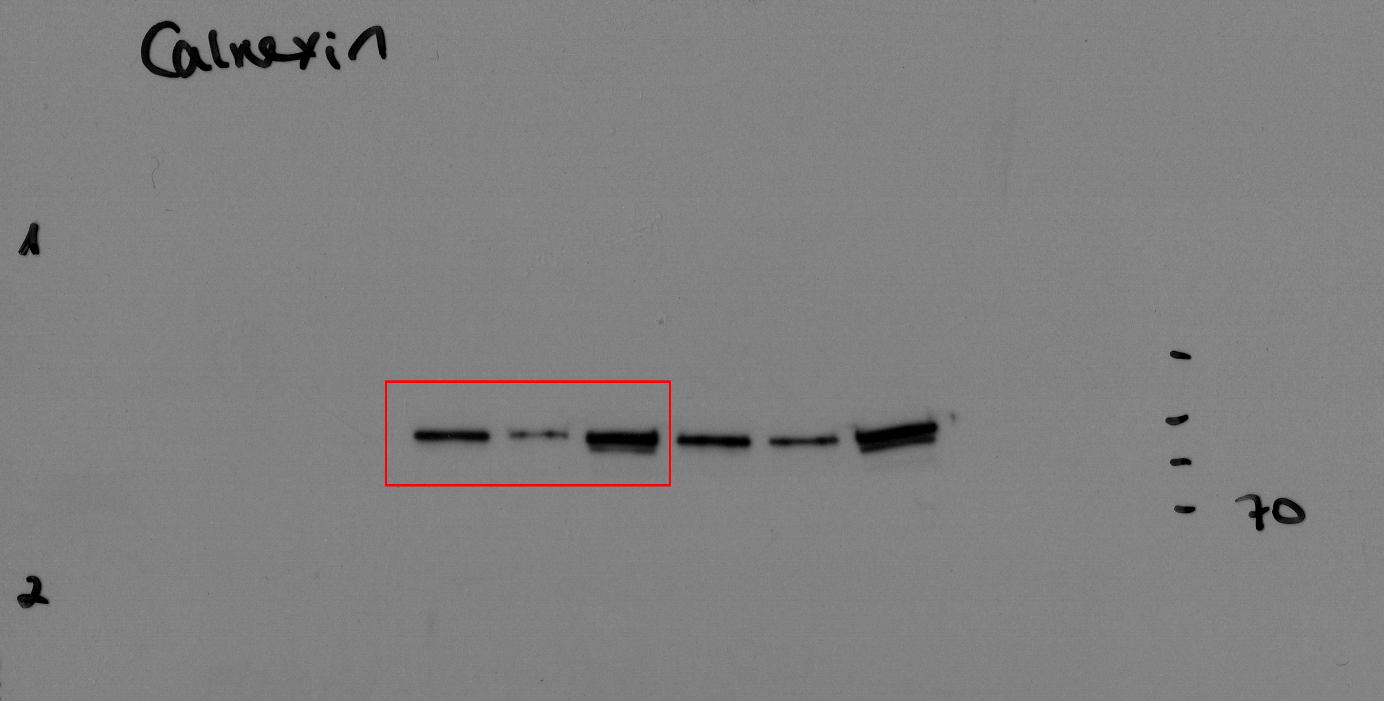
**

Fig.5 H: Gapdh

**
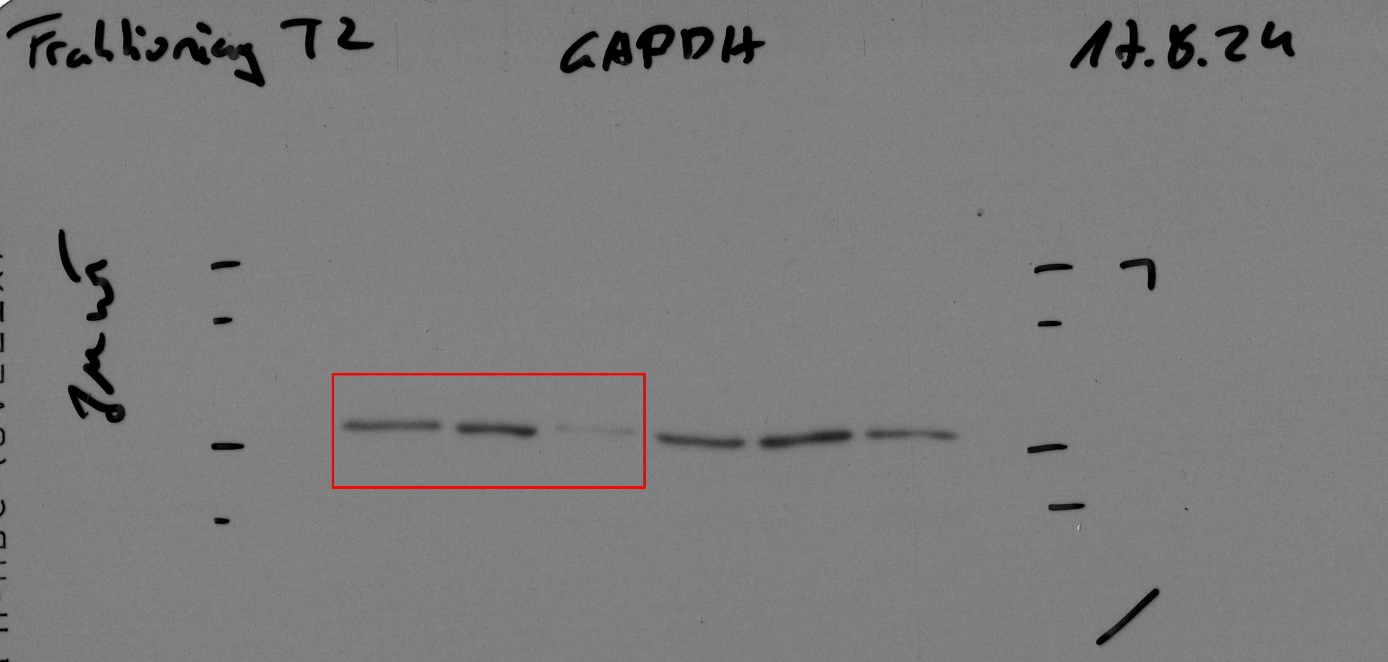
**

Fig.5 I: p-WIPI2 (LE)

**
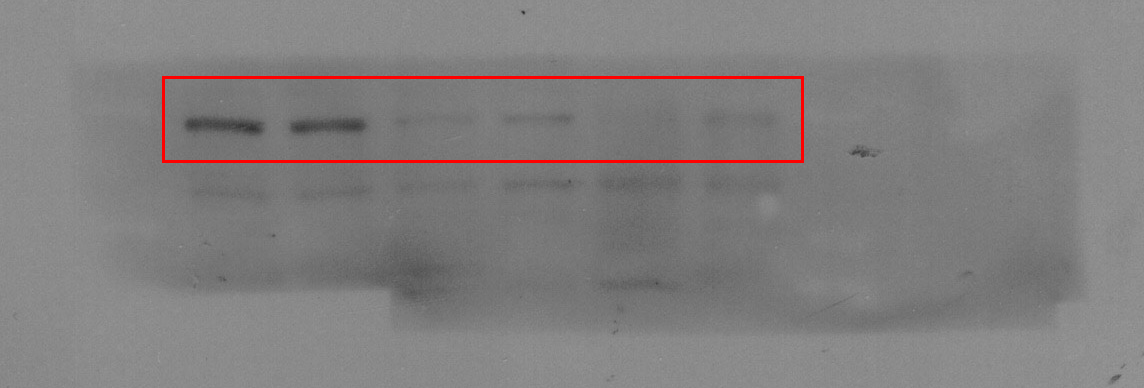
**

Fig.5 I: p-WIPI2 (HE)

**
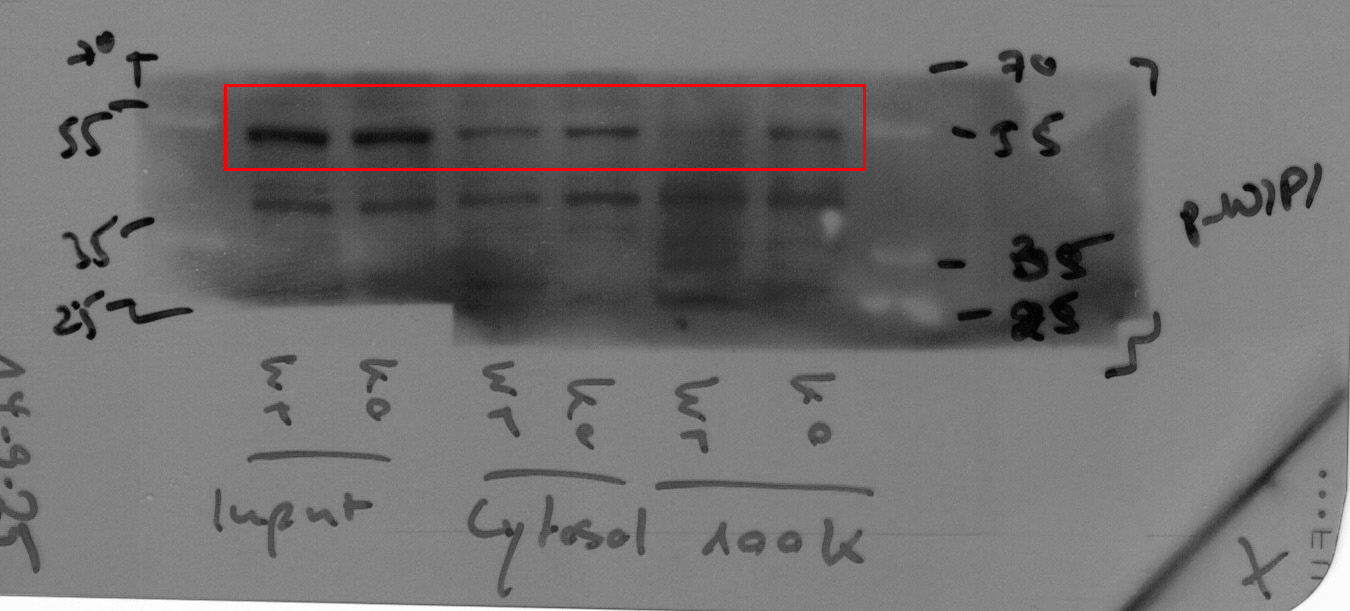
**

Fig.5 I: WIPI2


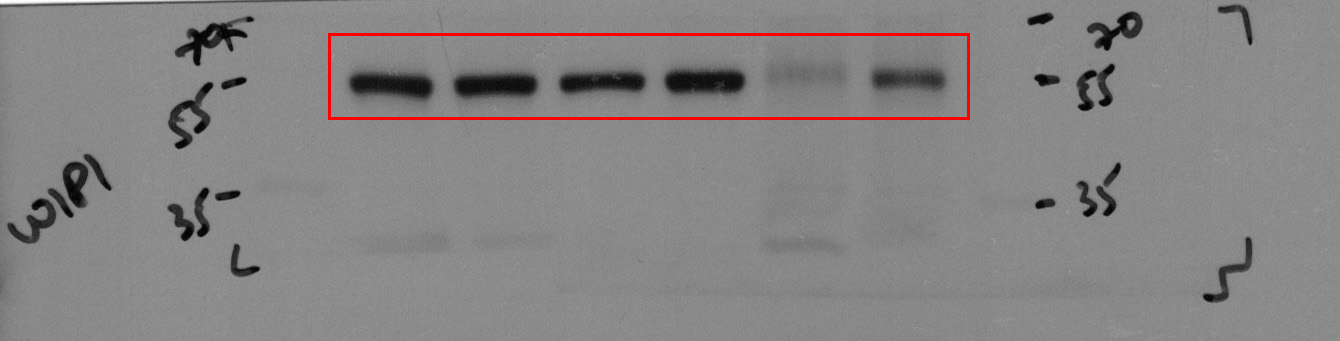


Fig.5 I: CNX


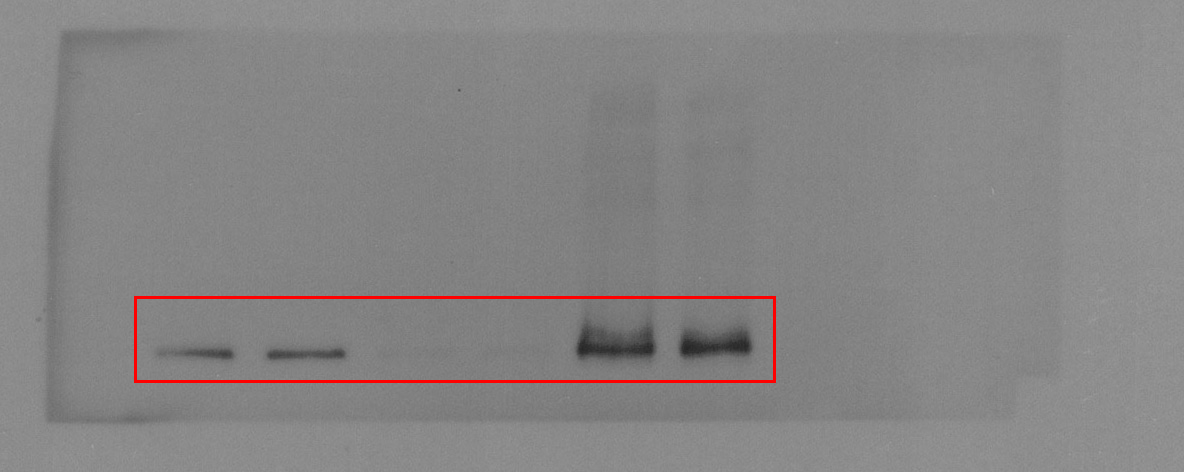


Fig.5 I: Gapdh


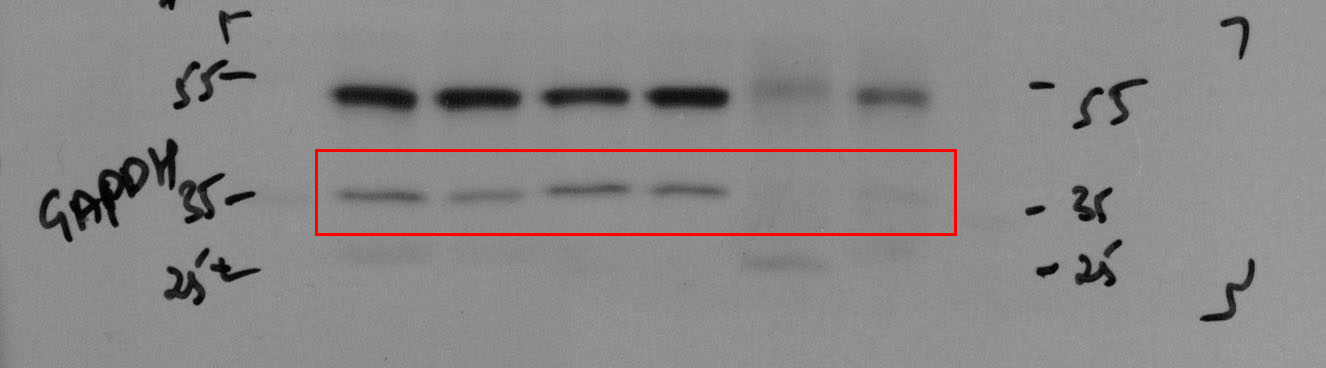


Fig.5 J: p-WIPI2 cytosol


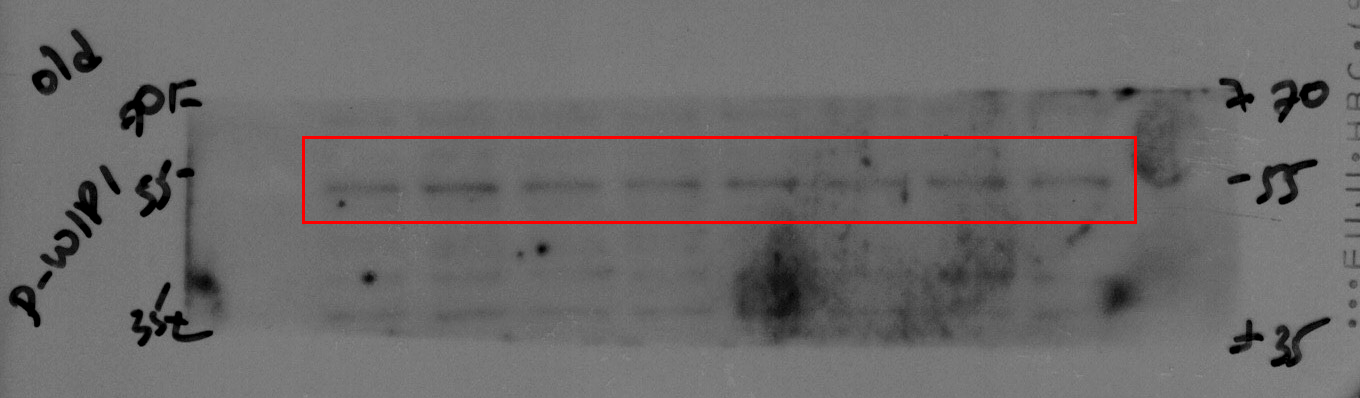


Fig.5 J: WIPI2 cytosol


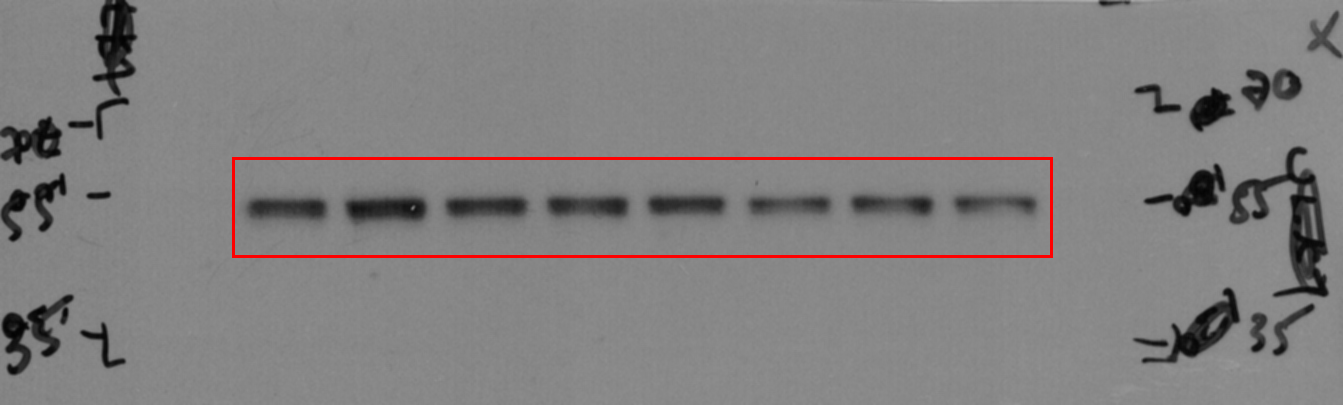


Fig.5 J: CNX cytosol


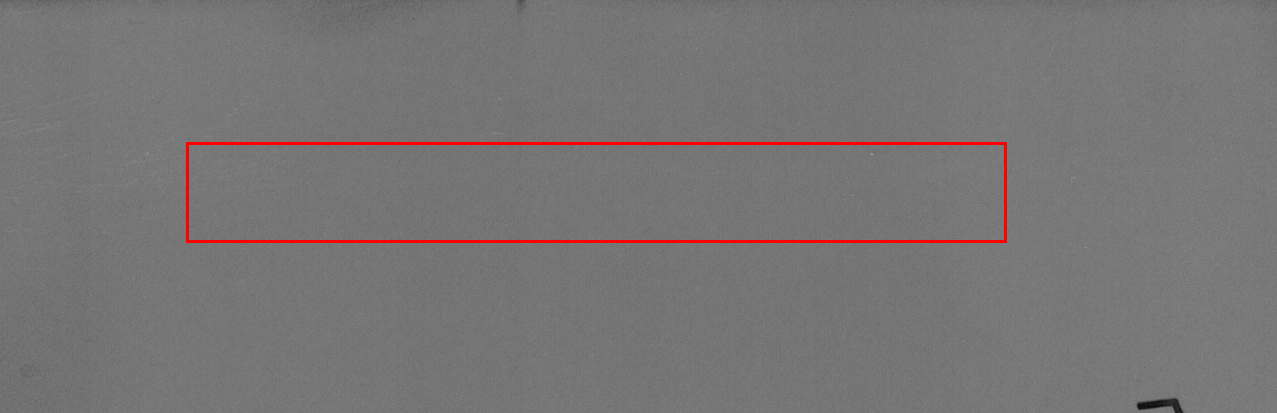


Fig.5 J: Gapdh cytosol


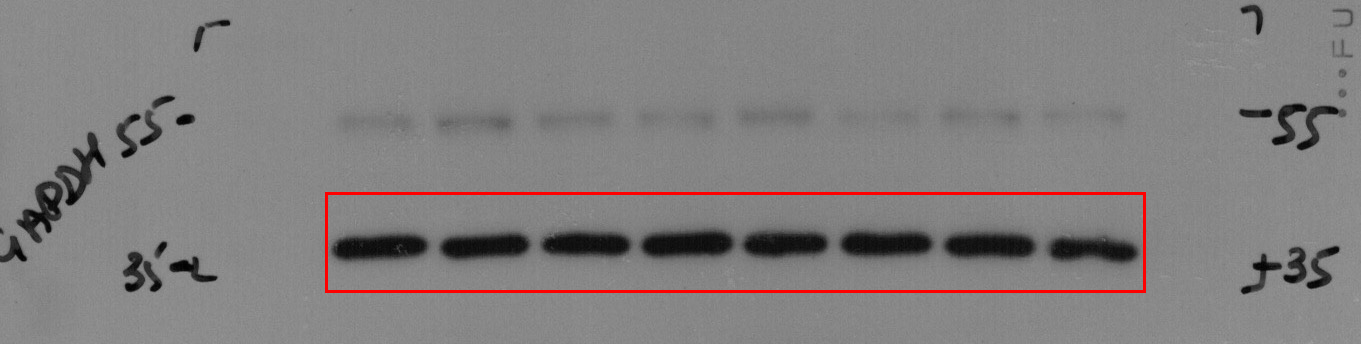


Fig.5 J: p-WIPI2 membrane


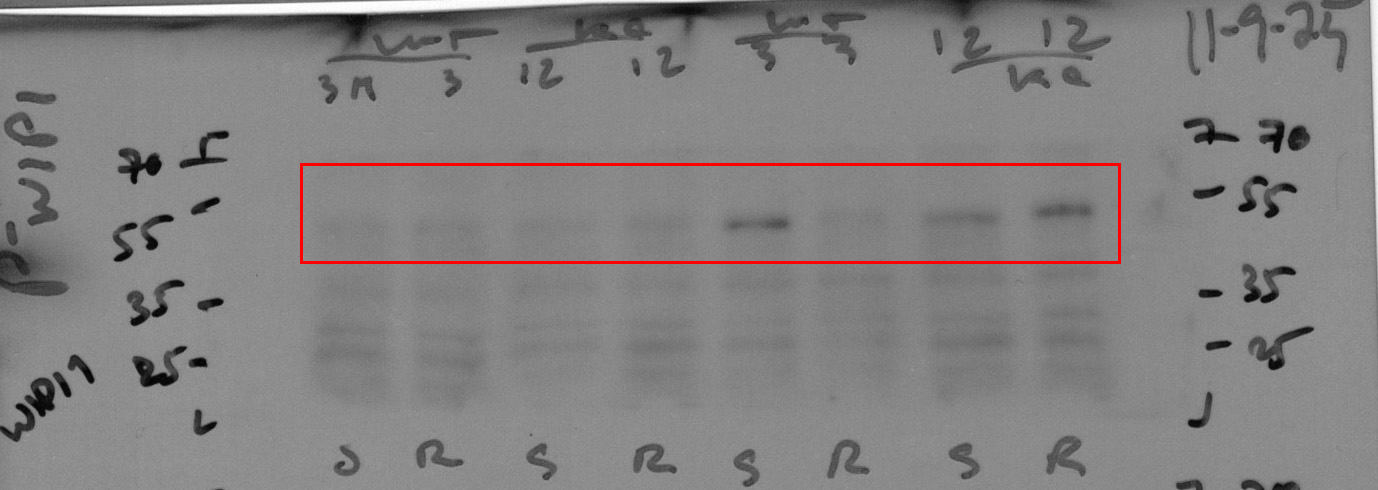


Fig.5 J: WIPI2 membrane


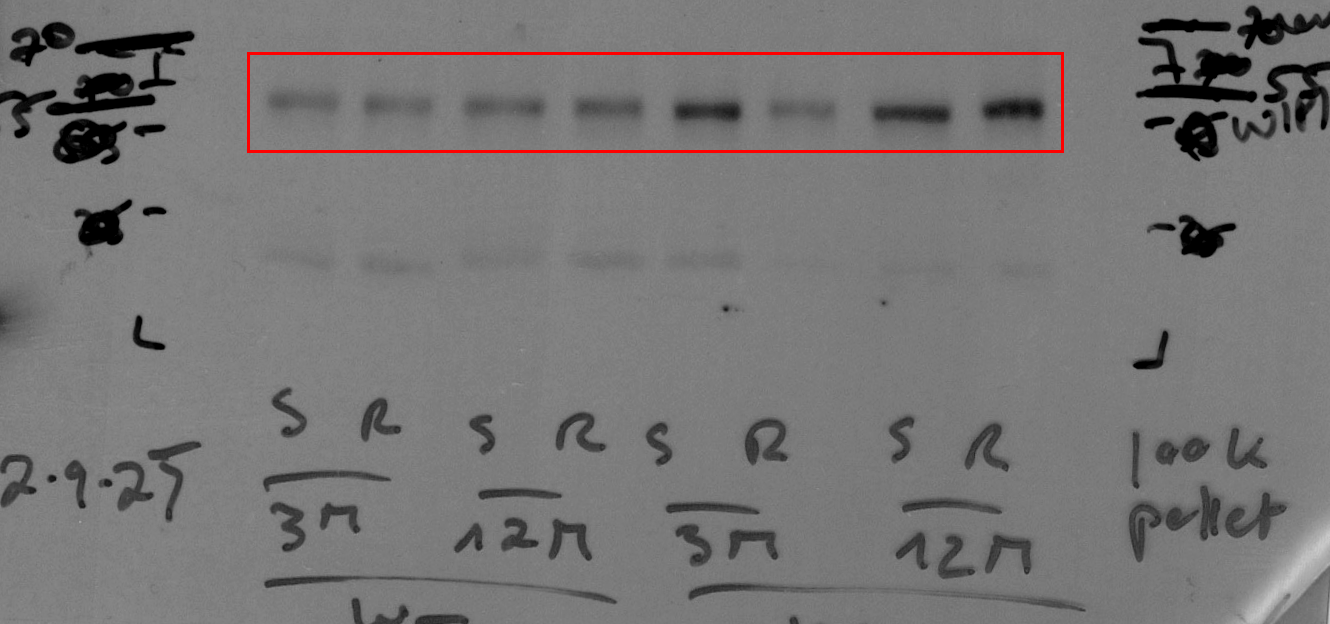


Fig.5 J: CNX membrane


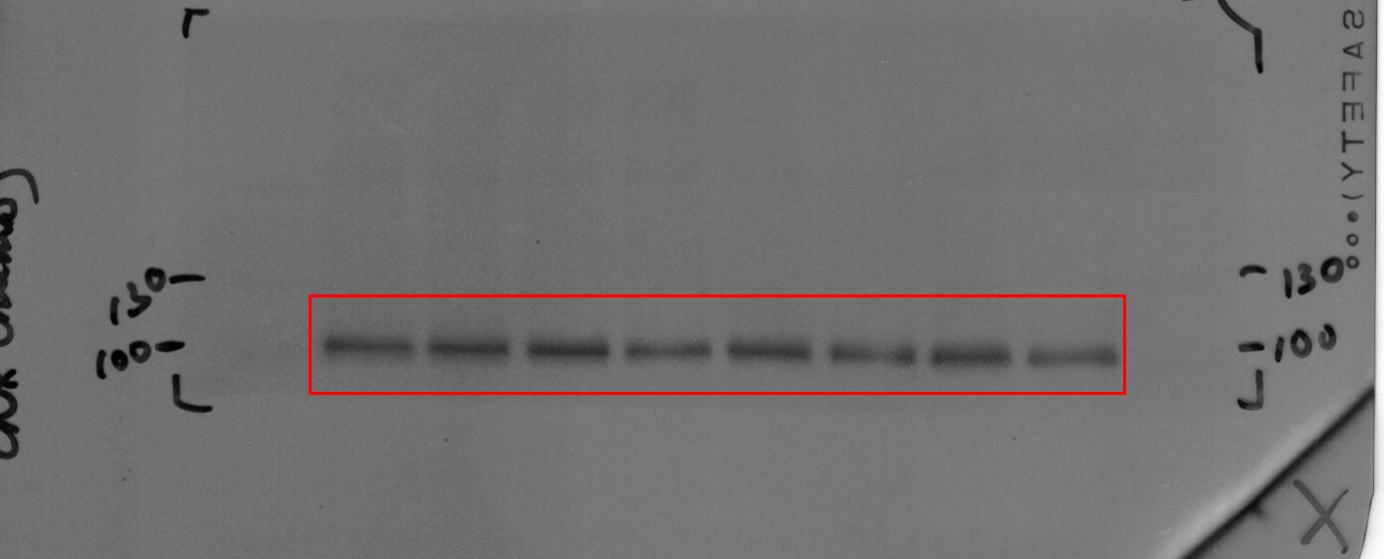


Fig.5 J: Gapdh membrane


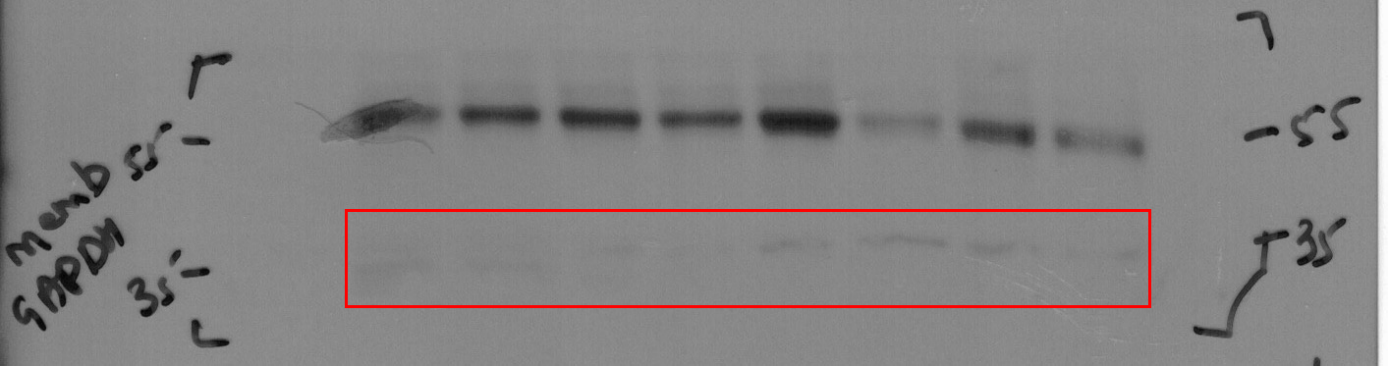


Sup.Fig.4 H: Atg9

**
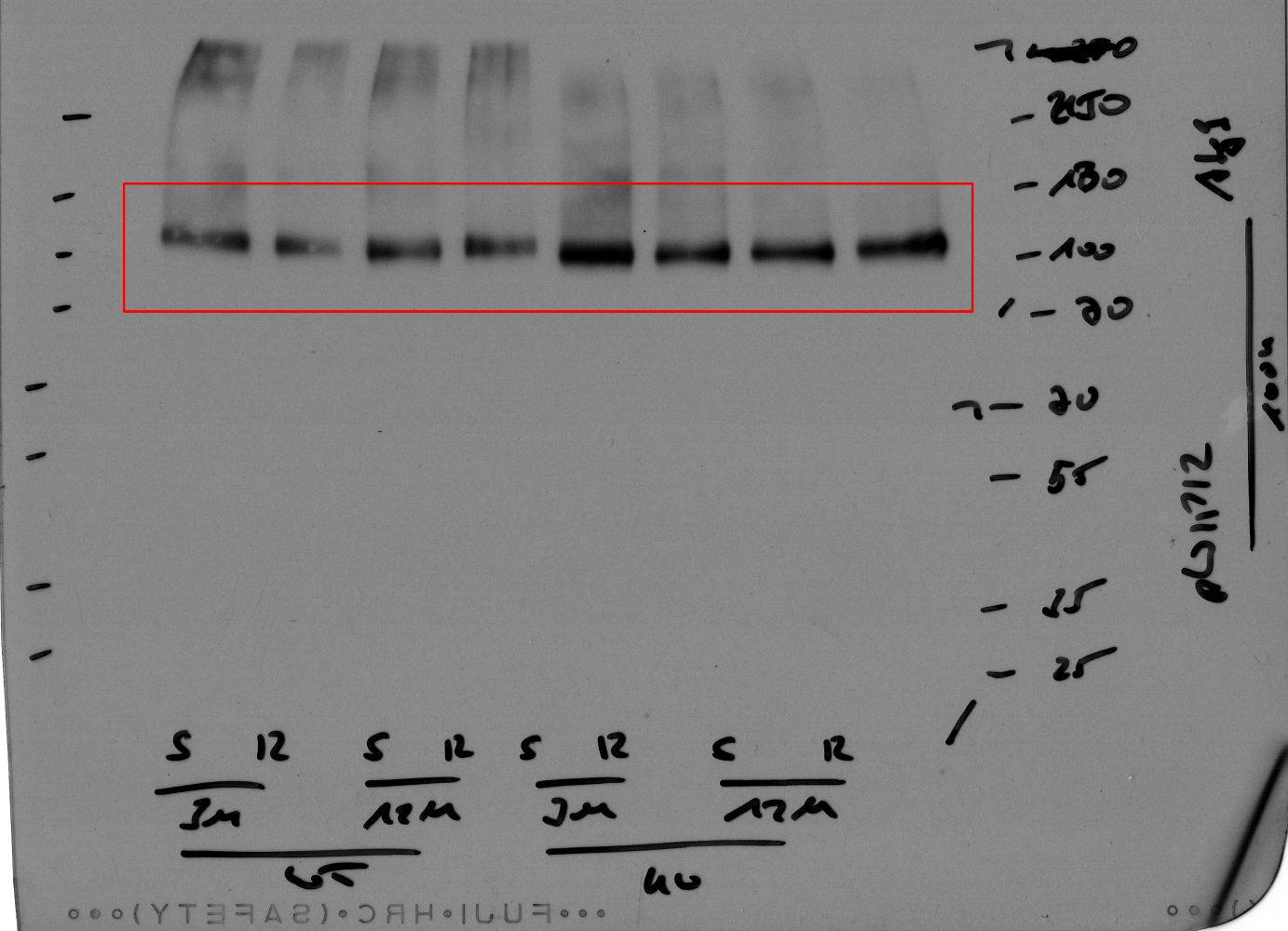
**

Sup.Fig.4 H: CNX

**
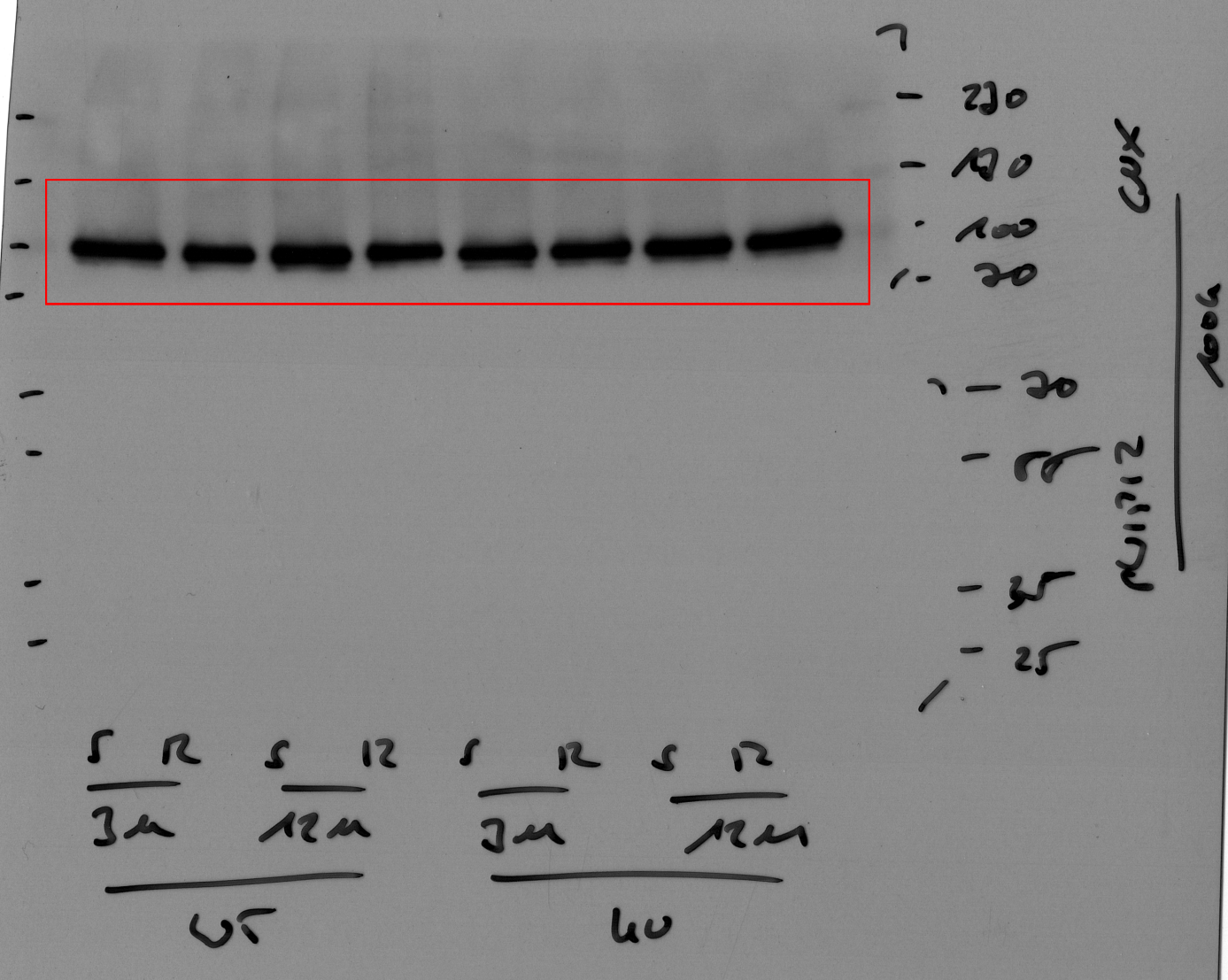
**
